# Early neuroimaging and delayed neurological sequelae in carbon monoxide poisoning: a systematic review and meta-analysis

**DOI:** 10.1038/s41598-022-07191-7

**Published:** 2022-03-03

**Authors:** Chiwon Ahn, Jaehoon Oh, Chan Woong Kim, Heekyung Lee, Tae Ho Lim, Hyunggoo Kang

**Affiliations:** 1 Department of Emergency Medicine, College of Medicine, Chung-Ang University, Seoul, Republic of Korea; 2 Department of Emergency Medicine, College of Medicine, Hanyang University, 222, Wangsimni-ro, Seongdong-gu, Seoul, 04763 Republic of Korea

**Keywords:** Diseases, Medical research, Neurology, Signs and symptoms

## Abstract

We aimed to assess the evidence regarding the usefulness of brain imaging as a diagnostic tool for delayed neurological sequelae (DNS) in patients with acute carbon monoxide poisoning (COP). Observational studies that included adult patients with COP and DNS were retrieved from Embase, MEDLINE, and Cochrane Library databases in December 2020 and pooled using a random-effects model. Seventeen studies were systematically reviewed. Eight and seven studies on magnetic resonance imaging (MRI) and computed tomography (CT), respectively, underwent meta-analysis. The pooled sensitivity and specificity of MRI for diagnosis of DNS were 70.9% (95% confidence interval [CI] 64.8–76.3%, I^2^ = 0%) and 84.2% (95% CI 80.1–87.6%, I^2^ = 63%), respectively. The pooled sensitivity and specificity of CT were 72.9% (95% CI 62.5–81.3%, I^2^ = 8%) and 78.2% (95% CI 74.4–87.1%, I^2^ = 91%), respectively. The areas under the curve for MRI and CT were 0.81 (standard error, 0.08; Q* = 0.74) and 0.80 (standard error, 0.05, Q* = 0.74), respectively. The results indicate that detecting abnormal brain lesions using MRI or CT may assist in diagnosing DNS in acute COP patients.

## Introduction

Carbon monoxide poisoning (COP) is a leading cause of poisoning-related mortality worldwide^1^. Compared with oxygen, CO has a 250-fold greater affinity with hemoglobin, competitively combining with it to produce carboxyhemoglobin (COHb). COP symptoms are varied but they are also non-specific^2^. If clinical information related to poisoning cannot be assessed due to, for example, an altered mental state, COP may be missed or the diagnosis may be delayed.

After the onset of acute COP symptoms, some patients may develop delayed neurological sequelae (DNS) 2–40 days after CO exposure^3–5^. In some patients with acute COP, an asymptomatic lucid period may occur, after which DNS develops^3–5^. The COHb level measured in an emergency setting upon the onset of poisoning symptoms cannot diagnose DNS. Accordingly, clinicians are investigating several factors, including clinical manifestations, such as loss of consciousness (LOC) or a low score on the Glasgow Coma Scale (GCS)^2,6–9^, and blood biomarkers, such as elevated troponin or creatinine kinase levels^2,6,10^, that may predict the development of sequelae. Additionally, Liao et al. showed that QT prolongation on electrocardiogram combined with LOC and a low GCS score was associated with the occurrence of DNS^8^. However, other studies report conflicting results or poor evidence regarding the use of these factors as DNS diagnostics^7,11,12^. Thus, no validated tool for diagnosing DNS in the acute poisoning phase currently exists.

COP may induce hypoxic damage to the brain, which starts the demyelinating process of white matter that is recognized as the main pathologic feature of neurological^13–15^. Therefore, lesions caused by COP can be identified through brain imaging examinations during the acute poisoning phase. Particularly, magnetic resonance imaging (MRI) can sensitively recognize COP-related cytotoxic edema when performed within 72 h after exposure to CO^16^, demonstrating the possibility of diagnosing DNS in an early phase.

We aimed to identify studies that performed brain imaging examinations of patients during the acute phase of COP and use meta-analysis to determine the possibility of DNS diagnosis by abnormal lesions in early computed tomography (CT) and MRI.

## Results

### Study selection

**Figure 1:**
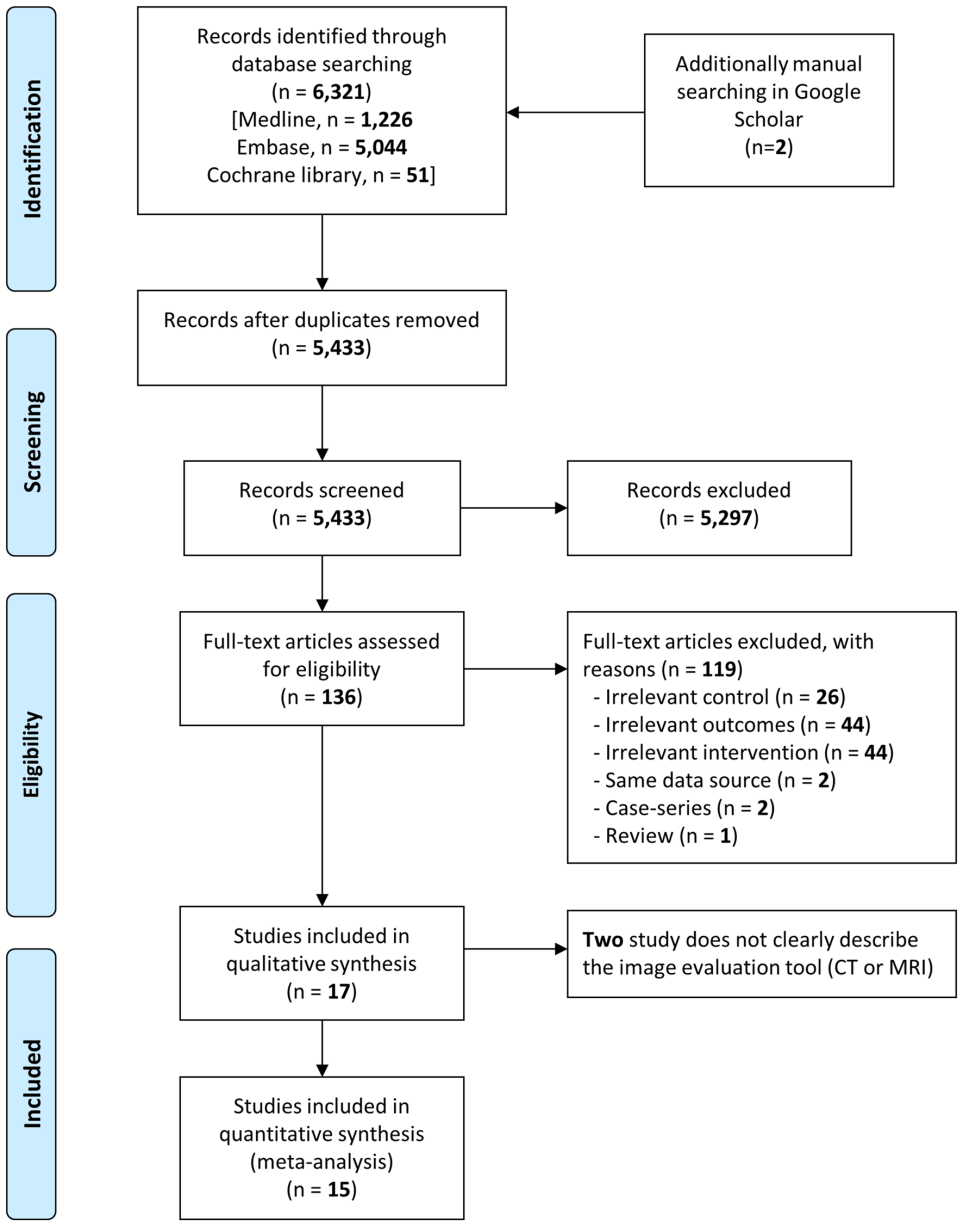
Flow diagram for identification of relevant studies on early neuroimage and delayed neurological sequelae in acute carbon monoxide poisoning.

The process used for identifying eligible studies is shown in Fig. 1. We excluded 119 studies because of irrelevant control groups (n = 26), outcome measures (n = 44), or interventions (n = 44); data duplication from the same trial (n = 2); and article type (case-series [n = 2] and review [n = 1]) (Supplementary Table S1). Finally, 17 studies including 2555 patients met the eligibility criteria and were included in this analysis (Supplementary Table S2).

### Study characteristics

MRI and CT examinations were performed in eight and seven studies, respectively, to diagnose DNS. Two studies did not specify the imaging technique used and were excluded from the meta-analysis. Fifteen studies were conducted in East Asia and two studies were conducted in Turkey and Egypt. One study was a multicenter investigation, while the remaining studies were single-center investigations. The maximum follow-up period of the analyzed studies ranged from 6 weeks to 6 months. The characteristics of the included studies are summarized in Table 1.

**Table 1:** Characteristics of all 17 studies included in systematic review.

Study	Region	Period	Design	Inclusion criteria	Timing of imaging examination	Number of pts. (DNS/no DNS)	Age	Male	HBOT	Maximum time of assessment of DNS
**MRI**										
Kim 2020	Korea	Nov 2016–Sep 2019	Retrospective Single	Acute COP	Within 240 h of the last CO exposure	9/40	43.0 (29.0–54.0)	64 (62.1%)	103 (100.0%)	6 weeks
Kokulu 2020	Turkey	Aug 2018–Jul 2019	Prospective Single	COHb ≥ 5% (Smokers: ≥ 10%)	As soon as possible after presentation to the ED	54/129	38.0 (28.0–53.0)	110 (60.1%)	116 (63.4%)	6 weeks
Lee 2020	Korea	Jan 2018–Jul 2018	Retrospective Single	COHb ≥ 3% (Smokers: ≥ 10%)	At the acute poisoning phase	12/126	36 (26–52)	75 (54.3%)	129 (93.5%)	6 weeks
Nah 2020	Korea	Aug 2016–Jul 2019	Prospective Single	COHb ≥ 5% (Smokers: ≥ 10%)	Within 2 days of visiting the ED	30/124	40.8	101 (65.6%)	154 (100.0%)	3 months
Jeon 2018	Korea	Apr 2011–Dec 2015	Prospective Single	Acute COP	Within hours of visiting the ED	101/286	42.0 (32.0–56.0)	244 (63.0%)	356 (92.0%)	6 weeks
Kim 2018	Korea	Jan 2015–May 2016	Retrospective Single	COHb ≥ 5% (Smokers: ≥ 10%)	Within 72 h after CO exposure	10/92	55.5 (36.8–69)	59 (57.8%)	97 (95.1%)	2 months
Kitamoto 2016	Japan	Jan 2006–Dec 2012	Retrospective Single	Acute COP	Within 3 days of CO exposure	11/69	45.9 ± 15.6	64 (80.0%)	0 (0%)	42 days or more
Park 2012	Korea	Mar 2011–Sep 2011	Retrospective Single	COHb ≥ 3% (Smokers: ≥ 10%)	Not reported	10/61	34.4 ± 14.1	51 (71.8%)	15 (21.1%)	Not reported
**CT**										
Du 2019	China	Jan 2013–Jan 2016	Retrospective Single	Acute COP	Within 24 h of admission to hospital	27/96	45.2	50 (40.7%)	NR	60 days
Liao 2019	Chinese Taipei	Jan 2009–Dec 2015	Retrospective Single	COHb ≥ 5% (Smokers: ≥ 10%)	At first medical institution	48/231	34.2 ± 17.0	130 (46.6%)	279 (100.0%)	42 days
Tianhong 2018	China	2008–2016	Retrospective Single	Acute COP with coma history	At the time of admission to the hospital from ED	36/148	46.7 ± 12.8	98 (53.3%)	184(100.0%)	90 days or more
Kudo 2014	Japan	2002–2011	Retrospective Single	Acute COP	At the acute poisoning phase	13/65	41.6	65 (82.3%)	49 (62.0%)	
Yang 2011	Chinese Taipei	May 2007–Oct 2008	Prospectively Single	COHb ≥ 10%	Within 5 days after acute COP	5/15	39.6	15 (55.6%)	27 (100.0%)	35 days
Ku 2010	Chinese Taipei	May 2005–Apr 2006	Retrospective Single	Acute COP	Within 5 days after acute COP	13/17	38.0 ± 11.1	26 (86.7%)	30 (100.0%)	6 months
Ide 2009	Japan	Nov 2006–Feb 2008	Retrospective Single	Acute COP	On admission	2/7	49.1 ± 8.6	5 (55.6%)	9 (100.0%)	3 months
**Brain image (do not distinguish of brain image type)**										
Gaballah 2020	Egypt	Jan 2018–Dec 2018	Retrospective Single	COHb ≥ 5% (Smokers: ≥ 10%)	At the time of admission	10/20	28.8 ± 13.1	19 (63.3%)	4 (13.3%)	6 months
Lin 2018	Chinese Taipei	Jan 1990–Dec 2011	Retrospective Multicenter	COHb ≥ 5% (Smokers: ≥ 10%)	At the emergency department	47/591	35.05 (16.27)	311 (48.7%)	175 (27.4%)	NA

*CO* carbon monoxide, *COHb* carboxyhemoglobin, *COP* carbon monoxide poisoning, *CT* computed tomography, *DNS* delayed neurologic sequelae, *NA* not applicable, *HBOT* hyperbaric oxygenation therapy.

### Quality of included studies

Among the 17 included studies, five fulfilled all quality criteria (Supplementary Table S3). In the index text, the assessment was performed with respect to the timing of imaging examinations. If the timing of imaging after visiting the emergency room was clear, it was deemed “low risk;” if the timing was unclear, it was deemed “unclear;” and if the timing was long after COP onset, it was deemed “high risk”. Four studies included items with a high risk-of-bias. Among those studies, three had three items with a high risk-of-bias. If ≥ 5 of the seven domains were deemed low in bias, the overall quality of the study was considered high (low bias); otherwise, the study was considered of low quality (high bias). Among the eight articles on MRI, six were considered high quality. Contrastingly, among the seven articles on CT, only two were considered high quality. The details of the quality assessments are available in Supplementary Table S3.

### MRI and CT for diagnosis of delayed neurological sequelae

**Figure 2:**
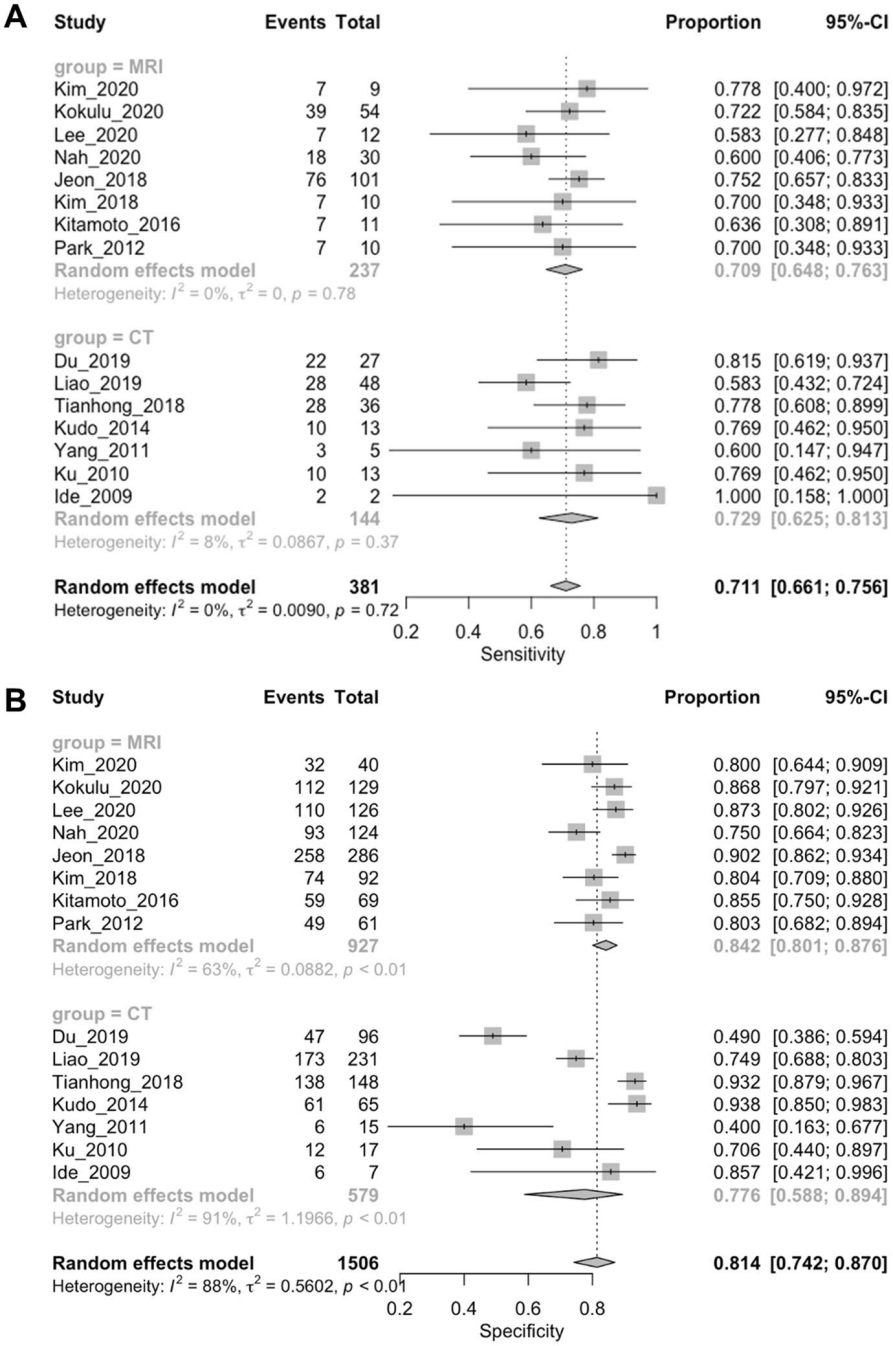
Forest plot for the sensitivity and specificity of MRI and CT for DNS diagnosis. (**A**) Sensitivity; (**B**) Specificity.

The sensitivity of MRI in detecting abnormal brain lesions ranged from 0.58 to 0.78, with a pooled value of 0.71 (95% CI 0.65–0.77) and low heterogeneity (I^2^ = 0.0%). The specificity ranged from 0.75 to 0.90, with a pooled value of 0.85 (95% CI 0.82–0.87) and high heterogeneity (I^2^ = 63.5%) (Fig. 2 and Table 2).

**Table 2:** Pooled estimates for prognostic accuracy of image with abnormal lesions for DNS diagnosis.

Type of imaging	Study, n	Pooled SEN (95% CI)^a^	Pooled SPE (95% CI)^a^	Pooled PLR (95% CI)	Pooled NLR (95% CI)	Pooled diagnostic OR (95% CI)^b^	Pooled symmetric AUC (SE)^c^	Q* (SE)^c^
MRI	8	0.709 (0.647–0.766)	0.849 (0.824–0.871)	4.278 (3.093–5.918)	0.357 (0.293–0.436)	12.091 (7.115–20.549)	0.808 (0.077)	0.743 (0.068)
CT	7	0.729 (0.634–0.787)	0.766 (0.729–0.800)	3.371 (1.755–6.473)	0.387 (0.257–0.581)	9.524 (3.301–20.480)	0.801 (0.050)	0.737 (0.044)

*AUC* area under curve, *CI* confidence interval, *CT* computed tomography, *MRI* magnetic resonance imaging, *NLR* negative likelihood ratio, *OR* odds ratio, *PLR* positive likelihood ratio, Q* the maximum joint sensitivity and specificity on a symmetric summary receiver operating characteristic curve, *SEN* sensitivity, *SPE* specificity.

^a^Presented as a forest plot in Fig. 2.

^b^Presented as a forest plot in Supplemental Fig. S1.

**Figure 3:**
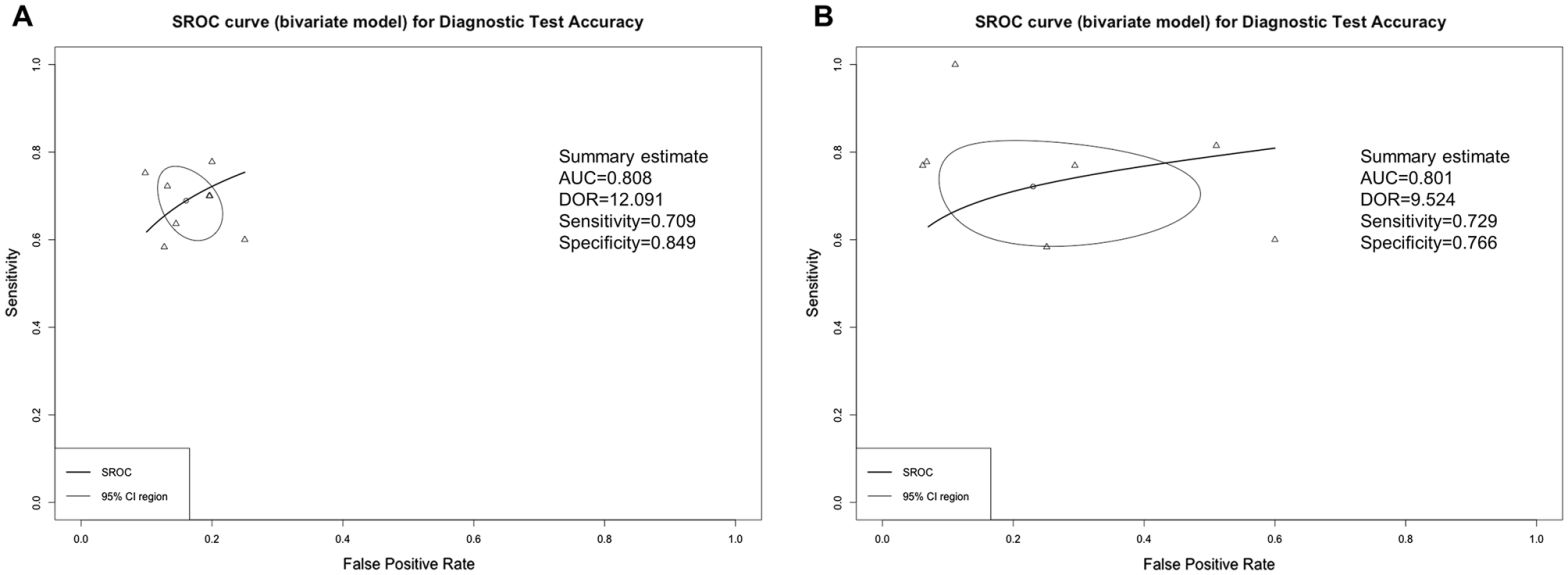
Summary receiver operating characteristic curves of MRI and CT for DNS diagnosis. (**A**) MRI; (**B**) CT. The curve is the regression line that summarized the overall prognostic accuracy. The upper and lower curves represent confidence intervals. Circles indicate individual study estimates of sensitivity and 1-specificity. The size of each circles is proportional to the sample size of individual study. *SROC* summary receiver operating characteristic, *AUC* area under the ROC curve, *DOR* diagnostic odd ratios.

^c^Presented as a summary receiver operating characteristic curve in Fig. 3.

The sensitivity of CT in detecting abnormal brain lesions ranged from 0.58 to 1.00, with a pooled value of 0.72 (95% CI 0.63–0.79) and moderate heterogeneity (I^2^ = 25.0%). The specificity ranged from 0.49 to 0.94, with a pooled value of 0.77 (95% CI 0.73–0.80) and high heterogeneity (I^2^ = 93.2%) (Fig. 2 and Table 2).

The prognostic accuracy assessed using summary receiver operating characteristic (SROC) analysis of MRI had an area under the curve (AUC) value of 0.81 (standard error [SE], 0.08; maximum joint sensitivity and specificity on a symmetric SROC curve [Q*], 0.74) (Fig. 3A). The prognostic accuracy assessed using SROC analysis of CT had an AUC value of 0.80 (SE, 0.05; Q*, 0.74) (Fig. 3B). Detailed values for each study are shown in Supplemental Table S4.

The MRI pooled diagnostic OR value for DNS was 12.09 (95% CI 7.12–20.55), and the CT pooled diagnostic OR was 9.36 (95% CI 3.26–26.88) (Supplementary Fig. S1 and Table 2).

### Subgroup analyses

Additionally, we performed subgroup analyses of articles on MRI and CT according to sample size (> 100 participants vs. < 100 participants), inclusion criteria (any COHb level vs. clinical signs and symptoms), imaging examination timing (≤ 72 h vs. unclear), proportion of patients who underwent HBOT (≥ 80% vs. < 80%), and study quality (high vs. low) (Tables 3 and 4).

**Table 3:** Pooled estimates for prognostic accuracy of MRI with abnormal lesions for DNS diagnosis by subgroup.

Characteristics	N*	Pooled SEN (95% CI)	Pooled SPE (95% CI)	Pooled PLR (95% CI)	Pooled NLR (95% CI)	Pooled DOR (95% CI)	Pooled symmetric AUC (SE)	Q (SE)
**Sample size**								
≥ 100	5	0.710 (0.644–0.769)	0.851 (0.825–0.875)	4.302 (2.861–6.470)	0.364 (0.284–0.467)	11.917 (6.201–22.904)	0.880 (0.117)	0.710 (0.099)
< 100	3	0.700 (0.506–0.853)	0.824 (0.758–0.878)	3.901 (2.614–5.821)	0.375 (0.217–0.647)	10.845 (4.489–26.200)	0.849 (0.078)	0.780 (0.073)
**Inclusion criteria**								
Any level of COHb	5	0.672 (0.579–0.757)	0.823 (0.788–0.855)	3.706 (2.668–5.146)	0.413 (0.318–0.537)	9.245 (5.319–16.067)	0.781 (0.103)	0.720 (0.088)
Clinical sign and symptom	3	0.744 (0.656–0.819)	0.884 (0.848–0.913)	5.526 (3.410–8.954)	0.293 (0.216–0.397)	22.980 (13.555–38.653)	0.864 (0.098)	0.795 (0.096)
**Timing of imaging examination**								
≤ 72 h	4	0.706 (0.627–0.777)	0.852 (0.822-.879)	4.195 (2.290–7.684)	0.391 (0.064–0.578)	10.797 (3.973–29.344)	0.756 (0.175)	0.698 (0.146)
Unclear	4	0.714 (0.605–0.808)	0.843 (0.797–0.882)	4.496 (3.312–6.102)	0.340 (0.242–0.478)	13.999 (7.791–25.155)	0.853 (0.076)	0.784 (0.072)
**Hyperbaric oxygen therapy**								
≥ 80%	5	0.710 (0.633–0.778)	0.849 (0.819–0.875)	4.145 (2.507–6.852)	0.379 (0.269–0.534)	11.263 (4.785–26.512)	0.810 (0.099)	0.745 (0.087)
< 80%	3	0.707 (0.590–0.806)	0.849 (0.800–0.891)	4.646 (3.312–6.516)	0.346 (0.243–0.493)	13.999 (7.517–26.072)	0.843 (0.141)	0.774 (0.131)
**Quality of study**								
High quality	6	0.706 (0.641–0.766)	0.855 (0.826–0.878)	4.437 (2.954–6.664)	0.369 (0.288–0.474)	12.023 (6.301–22.940)	0.773 (0.133)	0.713 (0.112)
Low quality	2	0.737 (0.488–0.909)	0.802 (0.711–0.875)	3.705 (2.293–5.985)	0.334 (0.157–0.711)	11.202 (3.596–34.892)	–	–

*AUC* area under curve, *CI* confidence interval, *CT* computed tomography, *MRI* magnetic resonance imaging, *NLR* negative likelihood ratio, *OR* odds ratio, *PLR* positive likelihood ratio, Q* the maximum joint sensitivity and specificity on a symmetric summary receiver operating characteristic curve, *SEN* sensitivity, *SPE* specificity.

*The reference list of included studies in each subgroup can be found in Supplemental Table S5.

**Table 4:** Pooled estimates for prognostic accuracy of CT with abnormal lesions for DNS diagnosis by subgroup.

Characteristics	N*	Pooled SEN (95% CI)	Pooled SPE (95% CI)	Pooled PLR (95% CI)	Pooled NLR (95% CI)	Pooled DOR (95% CI)	Pooled Symmetric AUC (SE)	Q (SE)
**Sample size**								
≥ 100	3	0.703 (0.609–0.786)	0.754 (0.712–0.792)	3.341 (1.319–8.465)	0.384 (0.214–0.690)	9.267 (2.035–42.204)	0.806 (0.098)	0.741 (0.086)
< 100	4	0.758 (0.577–0.889)	0.821 (0.734–0.888)	3.485 (1.125–10.800)	0.377 (0.191–0.747)	9.849 (1.638–59.221)	0.796 (0.058)	0.733 (0.050)
**Inclusion criteria**								
Any level of COHb	2	0.585 (0.441–0.719)	0.728 (0.667–0.782)	1.666 (0.743–3.736)	0.580 (0.417–0.807)	2.900 (0.854–9.846)	–	–
Clinical sign and symptom	5	0.791 (0.693–0.869)	0.794 (0.747–0.836)	4.911 (1.584–15.224)	0.279 (0.187–0.416)	17.534 (5.032–61.090)	0.862 (0.027)	0.793 (0.026)
**Hyperbaric oxygen therapy**								
≥ 80%	5	0.683 (0.584–0.771)	0.802 (0.761–0.839)	3.208 (1.396–7.377)	0.415 (0.239–0.719)	8.284 (2.028–33.833)	0.750 (0.082)	0.693 (0.068)
< 80%	2	0.800 (0.644–0.909)	0.671 (0.592–0.743)	4.247 (0.490–36.801)	0.318 (0.169–0.598)	13.499 (1.162–156.80)	–	–
**Quality of study**								
High quality	2	0.808 (0.642–0.942)	0.54 (0.424–0.622)	2.347 (0.743–7.412)	0.356 (0.163–0.774)	5.111 (1.663–15.704)	–	–
Low quality	5	0.687 (0.594–0.770)	0.819 (0.782–0.853)	3.810 (1.584–9.166)	0.392 (0.232–0.663)	10.315 (2.575–41.316)	0.729 (0.085)	0.677 (0.069)

*AUC* area under curve, *CI* confidence interval, *CT* computed tomography, *MRI* magnetic resonance imaging, *NLR* negative likelihood ratio, *OR* odds ratio, *PLR* positive likelihood ratio, Q* the maximum joint sensitivity and specificity on a symmetric summary receiver operating characteristic curve, *SEN* sensitivity, *SPE* specificity.

*The reference list of included studies in each subgroup can be found in Supplemental Table S5.

## Discussion

We evaluated the usefulness of neuroimaging to diagnose DNS in patients with COP who experienced lucid periods of improved acute poisoning symptoms, excluding patients with persistent neurological symptoms after COP. This meta-analysis of eight studies that used MRI to diagnose DNS in patients with COP showed that MRI had moderate sensitivity (0.71), high specificity (0.85) and an AUC value of 0.808, displaying better-than-moderate accuracy^17,18^. Accordingly, we suggested that abnormal brain lesions detected on MRI within 72 h of patient arrival to the emergency department may assist in diagnosing DNS in patients with acute COP.

COP may induce tissue inflammation and injury through ischemic reperfusion injury, CO-related vascular endothelium damage, oxygen radical-mediated lipid peroxidation, platelet-liberated nitric oxide^16,19–22^, and CO-induced cell death. The signs of COP-related damage in the brain white matter appear in imaging examinations such as CT or MRI. The progressive demyelination of deep cerebral white matter is a characteristic of DNS development in patients^23,24^. Jeon et al. showed that when MRI was performed within a few hours after CO exposure, DNS was diagnosed with a sensitivity and specificity of 75.2% and 90.2%, respectively^5^. In COP patients with DNS, similar brain imaging changes may occur due to the lucid period after symptom onset during the acute phase, in which the symptoms temporarily improve. Physicians may overlook the delayed neurologic deficit during the lucid interval but can diagnose DNS by identifying abnormal lesions through MRI in the acute COP phase.

Since there was no obvious consensus on the optimal test tool for diagnostic accuracy of DNS for patients with acute COP, various biomarker and tool were studied to detect the DNS in the early stage. Previous studies reported that LOC during the acute COP phase had diagnostic OR values ranging between 2.80 and 9.15^2,6,8,9^. Moreover, a GCS score < 9 during the acute poisoning phase had a diagnostic OR value of 7.15 (95% CI 1.04–44.8)^11^. Regarding biomarkers studied as potential DNS prediction factors, creatine kinase and troponin-I levels differed according to the presence or absence of DNS^2,6^. Chan et al. reported that a creatine kinase level > 3.33 µkat/L had a DNS diagnostic OR value of 6.0 (95% CI 0.66–54.77)^6^. Additionally, Pang et al. reported that plasma copeptin is highly related to the severity of COP and can diagnose DNS (diagnostic OR, 1.313; 95% CI 1.106–1.859)^25^. Finally, Liao et al. reported a diagnostic OR value of 3.86 (95% CI 2.22–6.70) for DNS occurrence based on QT prolongation on electrocardiogram examination^8^. However, other studies did not provide any meaningful results regarding abnormal electrocardiogram changes^6,7^.

Prominent and bright signal changes in abnormal lesions are evident on MRI, reflecting a series of damage caused by COP-induced ischemic changes. Additionally, the morphology, asymmetry, and location of the lesion must be determined^16,26^. Previous studies demonstrated that the globus pallidus structure represents the best area to observe abnormalities in patients with acute COP^5,27,28^. Jeon et al. observed abnormal findings in the globus pallidus in 19.9% of all patients with COP; most lesions were in the cortex, white matter hippocampus, and basal ganglia (including globus pallidus), whereas lesions were rarely observed in the brain stem and thalamus^5^. These findings are similar to those of lesions identified during the chronic COP phase and can be recognized using brain imaging examinations during the acute COP phase. Among the imaging techniques that may be performed during the acute phase of ischemic changes in the brain, MRI can recognize the final lesion more accurately and show the results with greater sensitivity than CT^29^. CT results are highly influenced by the professional’s ability to recognize hypoattenuation, and the technique’s sensitivity to recognize acute ischemic lesions is relatively low^29–32^. In this study, pooled diagnostic ORs were 12.09 (95% CI 7.12–20.55) and 9.5 (3.31–27.45) in MRI and CT, respectively, and I^2^ showed high heterogeneity at 77% in CT (In MRI, I^2^ 48%). Two studies included in meta-analysis of CT showed relatively high OR values of 48.30 (17.51–133.22) and 50.83 (9.89–261.92), which increased heterogeneity. Compared with the meta-analysis of MRI, most of those of CT are unclear at the time of imaging evaluation, or it is possible that the time was taken after the initial effect of COP progressed. In the very early stage, the change in the density of abnormal brain lesions was insignificant, so MRI could be more sensitive. However, as the CT scan is delayed, the DNS diagnosis rate is overestimated, and the unclear timing of the scan may have contributed to the increased heterogeneity between studies. Nevertheless, we could not compare the superiority between MRI and CT because this meta-analysis did not include a direct comparison between CT and MRI.

In the included studies, diffusion-weighted imaging was performed using 1.5-T or 3.0-T MRI scanners. Recently, diffusion kurtosis imaging (DKI) has emerged as an imaging technique with greater sensitivity to detect damages to the microstructure of the brain than diffusion-weighted or diffusion tensor imaging techniques^32,33^. DKI is a straightforward extension of the diffusion tensor imaging model and provides values for mean kurtosis. When abnormal lesions do not appear in the brain white and gray matters after COP onset, high mean kurtosis values strongly suggest poor prognosis in the delayed poisoning phase^34^. Despite the advantage of possibly identifying abnormal brain lesions at an earlier stage, few studies have confirmed the presence of abnormalities in DKI examinations of actual patients with COP. Therefore, this study could not include this modality as an MRI subgroup in the meta-analysis. Future studies should compare and analyze results using the DKI modality as a screening test in the MRI examination technique.

Previous studies have suggested that HBOT can prevent DNS and lower mortality owing to acute COP^35–37^. However, clinicians may consider age, sex, underlying diseases, and early symptoms when determining the benefit of the number HBOT sessions, because of the lack of standardized HBOT treatment protocols^37^. Additionally, MRI results obtained during the acute COP phase may influence this determination. MRI examination can be conducted within 24 h after performing one session of HBOT for patients with acute COP; thus, the presence of abnormal brain lesions can be combined with other factors to decide individual treatment protocols. Future studies should focus on recommending an HBOT protocol based on clinical evidence and including the presence or absence of brain lesions on MRI as a deciding factor.

There were several limitations. First, differences in the timing of imaging examination represented a significant limitation. Because changes in abnormal lesions detected on imaging examinations depends on imaging timing, these differences increase the heterogeneity of the included studies. Second, the generalizability of our results was limited because most included studies were geographically and ethnically/racially confined to East Asia. Our findings might have differed if the study included patients from other countries with different healthcare systems and ethnicities/races. Although a study has reported the relevance of ethnic/racial differences on mortality^38^, there is insufficient evidence of an association between ethnicity/race and DNS occurrence. Additional studies with wider representation are required to yield more robust conclusions. Third, a detailed analysis of HBOT including treatment variation, which may affect DNS occurrence, was not performed. An HBOT subgroup analysis was impossible because the included studies did not clearly describe this treatment’s use. Thus, our conclusions were limited because no detailed methods regarding oxygen therapy and HBOT were identified. Fourth, MRI could have been possibly performed during the onset of delayed symptoms after the lucid period. The minimum lucid interval period in the included studies ranged between 2–10 days. MRI was performed within a maximum of 72 h after CO exposure, but if symptoms of DNS appeared before conducting the brain imaging examination, bias could occur owing to the confirmatory rather than predictive nature of imaging in these situations. Finally, abnormal findings in CT could have been interpreted inconsistently. COP-induced abnormal brain lesions appear at low density on CT images and do not differ significantly from normal lesions during the acute poisoning phase, which may hinder their recognition.

In conclusion, the results indicate that detecting abnormal brain lesions using MRI or CT may assist in diagnosing DNS in acute COP patients. In addition, abnormal brain lesions detected on MRI within 72 h of patient arrival to the emergency department may assist in diagnosing DNS in patients with acute COP. Although CT also showed significant results in diagnosing DNS, changes in abnormal brain lesion density are subtle, limiting the effectiveness of CT as a predictive diagnostic technique.

## Methods

### Reporting guidelines and protocol registration

We conducted this review following the Preferred Reporting Items for Systematic Reviews and Meta-analysis guidelines to report information from randomized controlled trials^39^. Checklist was shown in Supplementary Table S6. The review protocol was prospectively registered in the PROSPERO database (CRD42020177002).

### Eligibility criteria

Observational studies were included. Reviews, case reports, editorials, letters, comments, conference abstracts, meta-analyses, and animal studies were excluded. The inclusion criteria were as follows: (1) studies including patients with COP who underwent brain imaging examination during the acute poisoning phase and (2) studies assessing the development of DNS during the subacute poisoning phase. The exclusion criteria were as follows: (1) studies including patients younger than 18 years, (2) those including patients who failed to recover from a decreased mental status (i.e., permanent neurologic injury) or died, and (3) non-original articles.

### Data sources and searches

Two experienced reviewers searched three electronic databases (MEDLINE, Embase, and Cochrane Library) for studies on early brain imaging to diagnose DNS in patients with COP published between January 1, 1970, and December 26, 2020. Search strategies included use of Medical Subject Headings (MeSH), Embase subject headings, and text words. We searched for additional studies in the reference lists of all identified studies, including relevant reviews. MeSH terms and free terms related to “carbon monoxide”, “carbon monoxide poisoning”, and “delayed neurological sequelae” were combined. The detailed study protocol and search strategies descriptions are provided in Supplementary Table S7.

### Study selection

According to certain pre-determined selection criteria, the reviewers independently screened the titles and abstracts of retrieved articles to exclude irrelevant studies and eliminate duplicate articles. If the title, authorship, and publication year were identical, the article was deemed a duplicate. Papers published with the same title and authorship but in distinct categories (i.e., published as a journal article and as a conference abstract) were deemed different. Subsequently, the reviewers conducted a full-text review of potentially relevant articles that met the inclusion criteria.

### Data extraction

The reviewers independently extracted the following information from the included studies: authors, year of publication, region of study, sample size, age, sex, type of imaging examination, administration of hyperbaric oxygen therapy (HBOT), and development of DNS. Discrepancies between reviewers were resolved by consensus. The main outcome extracted was the presence of pathology on brain imaging examination (MRI or CT) during the acute poisoning phase and subsequent development of DNS during the subacute poisoning phase. We extracted detailed data and generated a 2 × 2 table for each included study, from which results were pooled to measure the prognostic accuracy of brain imaging examinations for DNS development. The 2 × 2 tables were organized as follows: true positive = presence of pathology in brain imaging with DNS development; false positive = presence of pathology in brain imaging without DNS development; false positive = normal finding in brain imaging with DNS development, and false negative = normal finding in brain imaging without DNS development.

### Quality assessment

The methodological quality of each of the 17 selected studies was independently assessed by the reviewers with blinding to authorship and journal using the Quality Assessment of Diagnostic Accuracy Studies 2 tool^40^. Disagreement between reviewers was resolved by consensus. Four domains were evaluated: patient selection, index test, reference standard, and flow and timing. In the first three domains, risk-of-bias and applicability concerns were evaluated; in the last domain, only risk-of-bias was evaluated. The methodological quality of each selected study was categorized according to three parameters: “low risk-of-bias”, “high risk-of-bias”, and “unclear”.

### Statistical analyses

The primary analysis investigated the association between the presence of pathology on MRI and CT examinations during the acute poisoning phase and the subsequent development of DNS during the subacute poisoning phase. For dichotomous variables, pooled odds ratios (OR) with a 95% confidence interval (CI) were calculated using a random-effects model. The proportion of between-study inconsistency was estimated using the I^2^ statistic to assess heterogeneity; I^2^ values of 25%, 50%, and 75% were considered low, moderate, and high heterogeneity, respectively.

We conducted planned subgroup analyses based on sample sizes (≥ 100 vs. < 100), inclusion criteria (COHb level [≥ 5% or ≥ 3%] vs. clinical signs and symptoms), imaging examination timing (≤ 72 h vs. unclear), proportion of patients who underwent HBOT (≥ 80% vs. < 80%), and study quality (high vs. low). A sensitivity analysis was performed using sequential removal of individual studies and subsequent determination of an overall pooled approximation for the remaining studies. All meta-analyses were performed using R software (version 4.0.0, The R Foundation for Statistical Computing, Vienna, Austria) packages “meta” (version 4.11-0) and “metaphor” (version 2.1-0), and Meta-Disc software 1.4 (Clinical Biostatistics, Ramony Cajal Hospital, Madrid, Spain). Quality assessments of the included studies were performed using RevMan (version 5.4, Cochrane Collaboration 2012, Nordic Cochrane Centre, Copenhagen, Denmark).

## Supplementary Information

Supplementary Figure S1.

Supplementary Tables.

## Data Availability

The datasets generated during the current study are available from the corresponding author on reasonable request.
