# Supplementary material for: Early neuroimaging and delayed neurological sequelae in carbon monoxide poisoning: a systematic review and meta-analysis
Source: Sci Rep. 2022 Mar 3;12:3529. doi: 10.1038/s41598-022-07191-7 (PMC8894334; doi:10.1038/s41598-022-07191-7)

**Early neuroimaging and delayed neurological sequelae in carbon monoxide poisoning: a systematic review and meta-analysis**

Chiwon Ahn, MD, PhD^1*^; Jaehoon Oh, MD, PhD^2*^; Chan Woong Kim, MD, PhD^1^; Heekyung Lee, MD^2^; Tae Ho Lim, MD, PhD^2^; Hyunggoo Kang, MD, PhD^2^

*These authors contributed equally to this study and are co-first authors

^1^Department of Emergency Medicine, College of Medicine, Chung-Ang University, Seoul, Korea

^2^Department of Emergency Medicine, College of Medicine, Hanyang University, Seoul, Korea

**Corresponding author**: Hyunggoo Kang, MD, PhD
Department of Emergency Medicine, College of Medicine, Hanyang University
222, Wangsimni-ro, Seongdong-gu, Seoul, 04763, Korea
Tel: +82-2-2290-8999
Fax: +82-2-2290-9280
E-mail: emer0905@gmail.com

**Supplementary Figure S1. Forest plot of pooled diagnostic odd ratios**


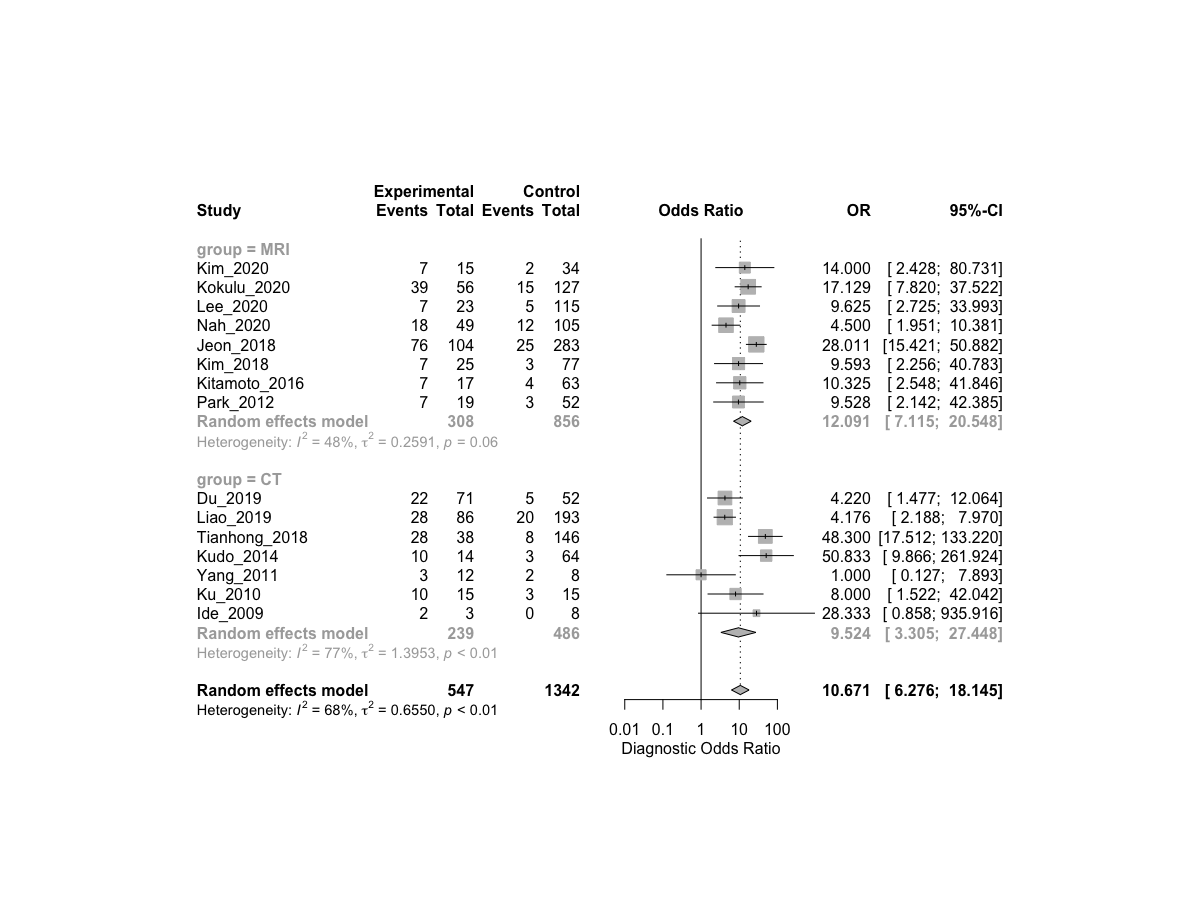

Supplement: Supplementary file 1 — Supplementary Figure S1. [file 41598_2022_7191_MOESM1_ESM.docx]
