# Supplementary material for: Early neuroimaging and delayed neurological sequelae in carbon monoxide poisoning: a systematic review and meta-analysis
Source: Sci Rep. 2022 Mar 3;12:3529. doi: 10.1038/s41598-022-07191-7 (PMC8894334; doi:10.1038/s41598-022-07191-7)
Supplement: Supplementary file 2 — Supplementary Tables. [file 41598_2022_7191_MOESM2_ESM.docx]

**Early neuroimaging and delayed neurological sequelae in carbon monoxide poisoning: a systematic review and meta-analysis**

Chiwon Ahn, MD, PhD^1†^; Jaehoon Oh, MD, PhD^2†^; Chan Woong Kim, MD, PhD^1^; Heekyung Lee, MD^2^; Tae Ho Lim, MD, PhD^2^; Hyunggoo Kang, MD, PhD^2^

^1^Department of Emergency Medicine, College of Medicine, Chung-Ang University, Seoul, Republic of Korea

^2^Department of Emergency Medicine, College of Medicine, Hanyang University, Seoul, Republic of Korea

^†^These authors contributed equally to this study and are co-first authors

**Corresponding author**: Hyunggoo Kang, MD, PhD
Department of Emergency Medicine, College of Medicine, Hanyang University
222, Wangsimni-ro, Seongdong-gu, Seoul, 04763, Republic of Korea
Tel: +82-2-2290-8999
Fax: +82-2-2290-9280
E-mail: emer0905@gmail.com

**Supplementary Table S1. List of excluded references after full-text review**

| **Number** | **Title** | **First author** | **Journal (Year)** | **Main reason for exclusion** |
| --- | --- | --- | --- | --- |
| 1 | Longitudinal gray matter changes of the pain matrix in patients with carbon monoxide intoxication: A voxel-based morphometry study | Chou MC et al. | European Journal of Radiology (2020) | DNS was not described. |
| 2 | S-100beta in predicting the need of hyperbaric oxygen in CO-induced delayed neurological sequels | Hafez AS et al. | Human & Experimental Toxicology (2020) | Intervention did not meet the criteria related to the brain imaging. |
| 3 | Exposure Duration and History of Hypertension Predicted Neurological Sequelae in Patients with Carbon Monoxide Poisoning | Huang CC et al. | Epidemiology (2019) | Intervention did not meet the criteria related to the brain imaging. |
| 4 | Carbon monoxide poisoning-induced delayed encephalopathy accompanies decreased microglial cell numbers: Distinctive pathophysiological features from hypoxemia-induced brain damage | Sekiya K et al. | Brain Research (2019) | Intervention did not meet the criteria related to the brain imaging. |
| 5 | Evaluation of changes in magnetic resonance diffusion tensor imaging after treatment of delayed encephalopathy due to carbon monoxide poisoning | Wu J et al. | Journal of Integrative Neuroscience (2019) | Control groups were not eligible for comparison with intervention groups. |
| 6 | Gray matter nuclei damage in acute carbon monoxide intoxication assessed in vivo using diffusion tensor MR imaging | Jiang W et al. | Radiologia Medica (2020) | DNS was not described. |
| 7 | Serum lactate as a predictor of neurologic outcome in ED patients with acute carbon monoxide poisoning | Jung JW et al. | American Journal of Emergency Medicine (2019) | DNS was not described. |
| 8 | Cerebral Damage after Carbon Monoxide Poisoning: A Longitudinal Diffusional Kurtosis Imaging Study | Zhang Y et al. | American Journal of Neuroradiology (2019) | DNS was not described. |
| 9 | The impact of hyperthermia after acute carbon monoxide poisoning on neurological sequelae | Moon JM et al. | Human & Experimental Toxicology (2019) | Intervention did not meet the criteria related to the brain imaging. |
| 10 | Clinical Predictors of Acute Brain Injury in Carbon Monoxide Poisoning Patients With Altered Mental Status at Admission to Emergency Department | Kim YJ et al. | Academic Emergency Medicine (2019) | DNS was not described. |
| 11 | Predictive Role of QTc Prolongation in Carbon Monoxide Poisoning-Related Delayed Neuropsychiatric Sequelae | Liao SC et al. | BioMed Research International (2018) | There was another literature about same trial. |
| 12 | Neuroprotective effect of ethanol in acute carbon monoxide intoxication: A retrospective study | Kim HH et al. | Medicine (2018) | DNS was not described. |
| 13 | Serum neuron-specific enolase levels at presentation and long-term neurological sequelae after acute charcoal burning-induced carbon monoxide poisoning | Moon JM et al. | Clinical Toxicology (2018) | DNS was not described. |
| 14 | Neurotoxicity of carbon monoxide targets caudate-mediated dopaminergic system | Sun TK et al. | Neurotoxicology (2018) | DNS was not described. |
| 15 | Serum N-terminal proBNP, not troponin I, at presentation predicts long-term neurologic outcome in acute charcoal-burning carbon monoxide intoxication | Moon JM et al. | Clinical Toxicology (2018) | DNS was not described. |
| 16 | Initial diffusion-weighted MRI and long-term neurologic outcomes in charcoal-burning carbon monoxide poisoning | Moon JM et al. | Clinical Toxicology (2018) | DNS was not described. |
| 17 | Serum neuron-specific enolase as an early predictor of delayed neuropsychiatric sequelae in patients with acute carbon monoxide poisoning | Cha YS et al. | Human & Experimental Toxicology (2018) | Intervention did not meet the criteria related to the brain imaging. |
| 18 | Efficacy of N-Butylphthalide and Hyperbaric Oxygen Therapy on Cognitive Dysfunction in Patients with Delayed Encephalopathy After Acute Carbon Monoxide Poisoning | Xiang W et al. | Medical Science Monitor (2017) | Control groups were not eligible for comparison with intervention groups. |
| 19 | Combined application of dexamethasone and hyperbaric oxygen therapy yields better efficacy for patients with delayed encephalopathy after acute carbon monoxide poisoning | Xiang W et al. | Drug design, development & therapy (2017) | Control groups were not eligible for comparison with intervention groups. |
| 20 | MRI and clinical manifestations of delayed encephalopathy after carbon monoxide poisoning | Wang X et al. | Pakistan Journal of Pharmaceutical Sciences (2016) | This study is case-series. |
| 21 | Reversible Changes of Brain Perfusion SPECT for Carbon Monoxide Poisoning-Induced Severe Akinetic Mutism | Chen SY et al. | Clinical Nuclear Medicine (2016) | This study is case-series. |
| 22 | Increased Long-Term Risk of Dementia in Patients With Carbon Monoxide Poisoning: A Population-Based Study | Wong CS et al. | Medicine (2016) | DNS was not described. |
| 23 | Acute carbon monoxide poisoning in a regional hospital in Hong Kong: historical cohort study | Chan MY et al. | Hong Kong Medical Journal (2016) | Intervention did not meet the criteria related to the brain imaging. |
| 24 | Altered white matter metabolism in delayed neurologic sequelae after carbon monoxide poisoning: A proton magnetic resonance spectroscopic study | Kuroda H et al. | Journal of the Neurological Sciences (2016) | Intervention did not meet the criteria related to the brain imaging. |
| 25 | Delayed visual disturbances in carbon monoxide poisoning: Identification and evaluation | Stabile JR et al. | Undersea & Hyperbaric Medicine (2015) | DNS was not described. |
| 26 | Increased risk of Parkinson disease in patients with carbon monoxide intoxication: a population-based cohort study | Lai CY et al. | Medicine (2015) | Intervention did not meet the criteria related to the brain imaging. |
| 27 | Cognitive severity-specific neuronal degenerative network in charcoal burning suicide-related carbon monoxide intoxication: a multimodality neuroimaging study in Taiwan | Chen NC et al. | Medicine (2015) | DNS was not described. |
| 28 | Novel clinical grading of delayed neurologic sequelae after carbon monoxide poisoning and factors associated with outcome | Kuroda H et al. | Neurotoxicology (2015) | Intervention did not meet the criteria related to the brain imaging. |
| 29 | Lack of pupil reflex and loss of consciousness predict 30-day neurological sequelae in patients with carbon monoxide poisoning | Zou JF et al. | PLoS ONE (2015) | Intervention did not meet the criteria related to the brain imaging. |
| 30 | 18F-FP-(+)-DTBZ positron emission tomography detection of monoaminergic deficient network in patients with carbon monoxide related parkinsonism | Chang CC et al. | European Journal of Neurology (2015) | DNS was not described. |
| 31 | Neuropsychological performance in patients with carbon monoxide poisoning | Yeh ZT et al. | Applied Neuropsychology: Adult (2014) | DNS was not described. |
| 32 | Plasma copeptin as a predictor of intoxication severity and delayed neurological sequelae in acute carbon monoxide poisoning | Pang L et al. | Peptides (2014) | Intervention did not meet the criteria related to the brain imaging. |
| 33 | A positive Babinski reflex predicts delayed neuropsychiatric sequelae in Chinese patients with carbon monoxide poisoning | Zou JF et al. | BioMed Research International (2014) | Intervention did not meet the criteria related to the brain imaging. |
| 34 | The impacts of acute carbon monoxide poisoning on the brain: Longitudinal clinical and 99mTc ethyl cysteinate brain SPECT characterization of patients with persistent and delayed neurological sequelae | Tsai CF et al. | Clinical Neurology & Neurosurgery (2014) | Control groups were not eligible for comparison with intervention groups. |
| 35 | The role of S100B protein, neuron-specific enolase, and glial fibrillary acidic protein in the evaluation of hypoxic brain injury in acute carbon monoxide poisoning | Akdemir HU et al. | Human & Experimental Toxicology (2014) | DNS was not described. |
| 36 | A PARK2 polymorphism associated with delayed neuropsychological sequelae after carbon monoxide poisoning | Liang F et al. | BMC Medical Genetics (2013) | Intervention did not meet the criteria related to the brain imaging. |
| 37 | Neuroprotective effects of erythropoietin in patients with carbon monoxide poisoning | Pang L et al. | Journal of Biochemical & Molecular Toxicology (2013) | DNS was not described. |
| 38 | Diffusion tensor imaging for predicting the clinical outcome of delayed encephalopathy of acute carbon monoxide poisoning | Hou X et al. | European Neurology (2013) | Control groups were not eligible for comparison with intervention groups. |
| 39 | Detection of gray matter damage using brain MRI and SPECT in carbon monoxide intoxication: a comparison study with neuropsychological correlation | Chen NC et al. | Clinical Nuclear Medicine (2013) | DNS was not described. |
| 40 | Diffusion-weighted imaging improves prediction in cognitive outcome and clinical phases in patients with carbon monoxide intoxication | Chen NC et al. | Neuroradiology (2013) | Intervention did not meet the criteria related to the brain imaging. |
| 41 | Delayed parkinsonism after CO intoxication: evaluation of the substantia nigra with inversion-recovery MR imaging | Kao HW et al. | Radiology (2012) | DNS was not described. |
| 42 | Delayed neuropsychological sequelae after carbon monoxide poisoning: predictive risk factors in the Emergency Department. A retrospective study | Pepe G et al. | Scandinavian Journal of Trauma, Resuscitation & Emergency Medicine (2011) | Intervention did not meet the criteria related to the brain imaging. |
| 43 | 1H-magnetic resonance spectroscopy indicates damage to cerebral white matter in the subacute phase after CO poisoning | Beppu T et al. | Journal of Neurology, Neurosurgery & Psychiatry (2011) | Intervention did not meet the criteria related to the brain imaging. |
| 44 | Neuropsychiatric disorders and risk factors in carbon monoxide intoxication | Katirci Y et al. | Toxicology & Industrial Health (2011) | Intervention did not meet the criteria related to the brain imaging. |
| 45 | Factors affecting the prognosis of patients with delayed encephalopathy after acute carbon monoxide poisoning | Hu H et al. | American Journal of Emergency Medicine (2011) | DNS was not described. |
| 46 | Hyperbaric oxygen ameliorates delayed neuropsychiatric syndrome of carbon monoxide poisoning | Chang DC et al. | Undersea & Hyperbaric Medicine (2010) | Control groups were not eligible for comparison with intervention groups. |
| 47 | Longitudinal study of carbon monoxide intoxication by diffusion tensor imaging with neuropsychiatric correlation | Chang CC et al. | Journal of Psychiatry & Neuroscience (2010) | Control groups were not eligible for comparison with intervention groups. |
| 48 | Assessment of damage to cerebral white matter fiber in the subacute phase after carbon monoxide poisoning using fractional anisotropy in diffusion tensor imaging | Beppu T et al. | Neuroradiology (2010) | Intervention did not meet the criteria related to the brain imaging. |
| 49 | S-100beta and neuron-specific enolase levels in carbon monoxide-related brain injury | Cakir Z et al. | American Journal of Emergency Medicine (2010) | DNS was not described. |
| 50 | Elevated serum S100B protein and neuron-specific enolase levels in carbon monoxide poisoning | Yardan T et al. | American Journal of Emergency Medicine (2009) | DNS was not described. |
| 51 | Damage of white matter tract correlated with neuropsychological deficits in carbon monoxide intoxication after hyperbaric oxygen therapy | Chang CC et al. | Journal of Neurotrauma (2009) | Intervention did not meet the criteria related to the brain imaging. |
| 52 | Myelin basic protein in cerebrospinal fluid: a predictive marker of delayed encephalopathy from carbon monoxide poisoning | Ide T et al. | American Journal of Emergency Medicine (2008) | There was another literature about same trial. |
| 53 | Cognitive and affective outcomes of more severe compared to less severe carbon monoxide poisoning | Chambers CA et al. | Brain Injury (2008) | DNS was not described. |
| 54 | Carbon monoxide poisoning: risk factors for cognitive sequelae and the role of hyperbaric oxygen | Weaver LK et al. | American Journal of Respiratory & Critical Care Medicine (2007) | DNS was not described. |
| 55 | Delayed encephalopathy after carbon monoxide intoxication--long-term prognosis and correlation of clinical manifestations and neuroimages | Hsiao CL et al. | Acta Neurologica Taiwanica (2004) | Control groups were not eligible for comparison with intervention groups. |
| 56 | S100B protein in carbon monoxide poisoning: a pilot study | Brvar M et al. | Resuscitation (2004) | DNS was not described. |
| 57 | Diffusion-weighted MRI and 99mTc-HMPAO SPECT in delayed relapsing type of carbon monoxide poisoning: evidence of delayed cytotoxic edema | Chu K et al. | European Neurology (2004) | Control groups were not eligible for comparison with intervention groups. |
| 58 | Memory one month after acute carbon monoxide intoxication: a prospective study | Deschamps D et al. | Occupational & Environmental Medicine (2003) | Control groups were not eligible for comparison with intervention groups. |
| 59 | Parkinsonism after carbon monoxide poisoning | Choi IS et al. | European Neurology (2002) | Control groups were not eligible for comparison with intervention groups. |
| 60 | White matter hyperintensities and neuropsychological outcome following carbon monoxide poisoning | Parkinson RB et al. | Neurology (2002) | DNS was not described. |
| 61 | Verbal memory deficits associated with fornix atrophy in carbon monoxide poisoning | Kesler SR et al. | Journal of the International Neuropsychological Society (2001) | DNS was not described. |
| 62 | Severe carbon monoxide poisoning: outcome after hyperbaric oxygen therapy | Hawkins M et al. | British Journal of Anaesthesia (2000) | DNS was not described. |
| 63 | The magnetic resonance imaging appearances of the brain in acute carbon monoxide poisoning | O'Donnell P et al. | Clinical Radiology (2000) | DNS was not described. |
| 64 | Clinical outcome and magnetic resonance imaging of carbon monoxide intoxication. A long-term follow-up study | Pavese N et al. | Italian Journal of Neurological Sciences (1999) | DNS was not described. |
| 65 | Delayed movement disorders after carbon monoxide poisoning | Choi IS et al. | European Neurology (1999) | Control groups were not eligible for comparison with intervention groups. |
| 66 | Regional cerebral blood flow measurements with Xenon-CT in the prediction of delayed encephalopathy after carbon monoxide intoxication | Sesay M et al. | Acta Neurologica Scandinavica (1996) | Control groups were not eligible for comparison with intervention groups. |
| 67 | Delayed neuropsychologic sequelae after carbon monoxide poisoning: prevention by treatment with hyperbaric oxygen | Thom SR et al. | Annals of Emergency Medicine (1995) | Control groups were not eligible for comparison with intervention groups. |
| 68 | Evaluation of outcome of delayed neurologic sequelae after carbon monoxide poisoning by technetium-99m hexamethylpropylene amine oxime brain single photon emission computed tomography | Choi IS et al. | European Neurology (1995) | Control groups were not eligible for comparison with intervention groups. |
| 69 | Computed tomographic findings after acute carbon monoxide poisoning | Jones JS et al. | American Journal of Emergency Medicine (1994) | DNS was not described. |
| 70 | Neurological sequelae following carbon monoxide poisoning clinical course and outcome according to the clinical types and brain computed tomography scan findings | Lee MS et al. | Movement Disorders (1994) | Control groups were not eligible for comparison with intervention groups. |
| 71 | Technetium-99m HM-PAO SPECT in patients with delayed neurologic sequelae after carbon monoxide poisoning | Choi IS et al. | Journal of Korean Medical Science (1992) | Control groups were not eligible for comparison with intervention groups. |
| 72 | A brain syndrome associated with delayed neuropsychiatric sequelae following acute carbon monoxide intoxication | Min SK et al. | Acta Psychiatrica Scandinavica (1986) | Control groups were not eligible for comparison with intervention groups. |
| 73 | Delayed neurologic sequelae in carbon monoxide intoxication | Choi IS et al. | Archives of Neurology (1983) | Control groups were not eligible for comparison with intervention groups. |
| 74 | Cox regression model of prognostic factors for delayed neuropsychiatric sequelae in patients with acute carbon monoxide poisoning: A prospective observational study | Han S et al. | NeuroToxicology (2021) | Intervention did not meet the criteria related to the brain imaging. |
| 75 | Mechanism of delayed encephalopathy after acute carbon monoxide poisoning | Huang YQ et al. | Neural Regeneration Research (2020) | Intervention did not meet the criteria related to the brain imaging. |
| 76 | Glasgow Coma Scale is a better delayed neurological sequelae risk factor than neurological examination abnormalities in carbon monoxide poisoning | Xu P et al. | American Journal of Emergency Medicine (2020) | Intervention did not meet the criteria related to the brain imaging. |
| 77 | Selective Susceptibility of Oligodendrocytes to Carbon Monoxide Poisoning: Implication for Delayed Neurologic Sequelae (DNS) | Tian X et al. | Frontiers in Psychiatry (2020) | Intervention did not meet the criteria related to the brain imaging. |
| 78 | Study on brain structure network of patients with delayed encephalopathy after carbon monoxide poisoning: based on diffusion tensor imaging | Jiang W et al. | Nuroradiology (2020) | Intervention did not meet the criteria related to the brain imaging. |
| 79 | Abnormal degree centrality in delayed encephalopathy after carbon monoxide poisoning: a resting-state fMRI study | Wu K et al. | Neuroradiology (2020) | Control groups were not eligible for comparison with intervention groups. |
| 80 | The Cannabinoid WIN 55,212-2 Reduces Delayed Neurologic Sequelae After Carbon Monoxide Poisoning by Promoting Microglial M2 Polarization Through ST2 Signaling | Du JJ et al. | Journal of Molecular Neuroscience (2020) | Intervention did not meet the criteria related to the brain imaging. |
| 81 | Oligodendrocyte dysfunction and regeneration failure: A novel hypothesis of delayed encephalopathy after carbon monoxide poisoning | Guo D et al. | Medical Hypotheses (2020) | Intervention did not meet the criteria related to the brain imaging. |
| 82 | The efficacy of N-butylphthalide and dexamethasone combined with hyperbaric oxygen on delayed encephalopathy after acute carbon monoxide poisoning | Zhang J et al. | Drug Design, Development and Therapy (2020) | Intervention did not meet the criteria related to the brain imaging. |
| 83 | Assessment of serum glucose/potassium ratio as a predictor for delayed neuropsychiatric syndrome of carbon monoxide poisoning | Demirtaş E et al. | Human and Experimental Toxicology (2020) | Intervention did not meet the criteria related to the brain imaging. |
| 84 | Multicenter retrospective analysis of the risk factors for delayed neurological sequelae after acute carbon monoxide poisoning | Zhang Y et al. | American Journal of Emergency Medicine (2020) | Intervention did not meet the criteria related to the brain imaging. |
| 85 | Association between Neuron-Specific Enolase Gene Polymorphism and Delayed Encephalopathy after Acute Carbon Monoxide Poisoning | Xu L et al. | Behavioural Neurology (2020) | Intervention did not meet the criteria related to the brain imaging. |
| 86 | Hyperbaric Oxygen Therapy Did Not Prevent Delayed Neuropsychiatric Sequelae in a Prospective Observational Study With Propensity Score Matching in 224 Patients With Acute Carbon Monoxide Toxicity | Han S et al. | Journal of Emergency Medicine (2020) | Intervention did not meet the criteria related to the brain imaging. |
| 87 | LRCH1 polymorphisms linked to delayed encephalopathy after acute carbon monoxide poisoning identified by GWAS analysis followed by Sequenom MassARRAY® validation | Gu J et al. | BMC Medical Genetics (2019) | Intervention did not meet the criteria related to the brain imaging. |
| 88 | Longitudinal white matter changes following carbon monoxide poisoning: A 9-month follow-up voxelwise diffusional kurtosis imaging study | Chou MC et al. | American Journal of Neuroradiology (2019) | Intervention did not meet the criteria related to the brain imaging. |
| 89 | Demographic characteristics and delayed neurological sequelae risk factors in carbon monoxide poisoning | Sarı Doğan F et al. | American Journal of Emergency Medicine (2019) | Control groups were not eligible for comparison with intervention groups. |
| 90 | Impact of hyperbaric oxygen therapy on subsequent neurological sequelae following carbon monoxide poisoning | Huang CC et al. | Journal of Clinical Medicine (2018) | Intervention did not meet the criteria related to the brain imaging. |
| 91 | Diffusion kurtosis imaging as a neuroimaging biomarker in patients with carbon monoxide intoxication | Lee JJ et al. | NeuroToxicology (2018) | DNS was not described. |
| 92 | Dynamic changes and clinical significance of serum S100B protein and glial fibrillary acidic protein in patients with delayed encephalopathy after acute carbon monoxide poisoning | Di C et al. | Pakistan Journal of Medical Sciences (2018) | Intervention did not meet the criteria related to the brain imaging. |
| 93 | Efficacy of combined glucocorticoid and hyperbaric oxygen therapy against delayed encephalopathy after carbon monoxide poisoning, and its effect on expression of immune-associated cytokines | Li N et al. | Tropical Journal of Pharmaceutical Research (2018) | Intervention did not meet the criteria related to the brain imaging. |
| 94 | Variability in Treatment for Carbon Monoxide Poisoning in Japan: A Multicenter Retrospective Survey | Fujita M et al. | Emergency Medicine International (2018) | DNS was not described. |
| 95 | Copeptin levels in carbon monoxide poisoning | İrem G et al. | Turkish Journal of Medical Sciences (2017) | DNS was not described. |
| 96 | Efficacy of Combined XingZhi-YiNao Granules and Hyperbaric Oxygen Therapy for Cognition and Motor Dysfunction in Patients with Delayed Encephalopathy after Acute Carbon Monoxide Poisoning | Qin L et al. | Evidence-based Complementary and Alternative Medicine (2017) | Intervention did not meet the criteria related to the brain imaging. |
| 97 | Lp-PLA2 variants associated with delayed encephalopathy after acute carbon monoxide poisoning | Zhao N et al. | International Journal of Clinical and Experimental Medicine (2016) | Intervention did not meet the criteria related to the brain imaging. |
| 98 | Metabolic Covariant Network in Relation to Nigrostriatal Degeneration in Carbon Monoxide Intoxication-Related Parkinsonism | Chang CC et al. | Frontiers in Neuroscience (2016) | Intervention did not meet the criteria related to the brain imaging. |
| 99 | Ischemia-modified albumin levels in the prediction of acute critical neurological findings in carbon monoxide poisoning | Daş M et al. | Kaohsiung Journal of Medical Sciences (2016) | DNS was not described. |
| 100 | Can initial lactate levels predict the severity of unintentional carbon monoxide poisoning? | Doʇan NÖ et al. | Human and Experimental Toxicology (2015) | DNS was not described. |
| 101 | Predicting poor outcome in patients with intentional carbon monoxide poisoning and acute respiratory failure: A retrospective study | Shen CH et al. | Journal of Medical Sciences (Taiwan) (2015) | DNS was not described. |
| 102 | Increased risk of Parkinson disease in patients with carbon monoxide intoxication | Lai CY et al. | Medicine (United States) (2015) | Control groups were not eligible for comparison with intervention groups. |
| 103 | Neuropsychological outcome after carbon monoxide exposure following a storm: A case-control study | Pages B et al. | BMC Neurology (2014) | DNS was not described. |
| 104 | Fractional anisotropy in the centrum semiovale as a quantitative indicator of cerebral white matter damage in the subacute phase in patients with carbon monoxide poisoning: Correlation with the concentration of myelin basic protein in cerebrospinal fluid | Beppu T et al. | Journal of Neurology (2012) | Intervention did not meet the criteria related to the brain imaging. |
| 105 | Detecting damaged regions of cerebral white matter in the subacute phase after carbon monoxide poisoning using voxel-based analysis with diffusion tensor imaging | Fujiwara S et al. | Neuroradiology (2012) | Intervention did not meet the criteria related to the brain imaging. |
| 106 | Elevated S100B level in cerebrospinal fluid could predict poor outcome of carbon monoxide poisoning | Ide T et al. | American Journal of Emergency Medicine (2012) | Intervention did not meet the criteria related to the brain imaging. |
| 107 | The value of initial lactate in patients with carbon monoxide intoxication: In the emergency department | Moon JM et al. | Human and Experimental Toxicology (2011) | DNS was not described. |
| 108 | Hydrogen-rich saline reduces delayed neurologic sequelae in experimental carbon monoxide toxicity | Sun Q et al. | Critical Care Medicine (2011) | Intervention did not meet the criteria related to the brain imaging. |
| 109 | Tc99m-sestamibi thigh SPECT/CT images for noninvasive assessment of skeletal muscle injury in carbon monoxide intoxication with clinical and pathological correlation | Huang SH et al. | Clinical Nuclear Medicine (2011) | DNS was not described. |
| 110 | Clinical significance of the pallidoreticular pathway in patients with carbon monoxide intoxication | Chang CC et al. | Brain (2011) | DNS was not described. |
| 111 | White matter damage in carbon monoxide intoxication assessed in vivo using diffusion tensor MR imaging | Lin WC et al. | American Journal of Neuroradiology (2009) | Control groups were not eligible for comparison with intervention groups. |
| 112 | Comparison of SPECT findings and neuropsychological sequelae in carbon monoxide and organophosphate poisoning | Ozyurt G et al. | Clinical Toxicology (2008) | Control groups were not eligible for comparison with intervention groups. |
| 113 | Affective outcome following carbon monoxide poisoning: A prospective longitudinal study | Jasper BW et al. | Cognitive and Behavioral Neurology (2005) | This study is the review related to previous case series. |
| 114 | Is elevated plasma lactate a useful marker in the evaluation of pure carbon monoxide poisoning? | Benaissa ML et al. | Intensive Care Medicine (2003) | DNS was not described. |
| 115 | Quantitative PET scan findings in carbon monoxide poisoning: Deficits seen in a matched pair | Pinkston JB et al. | Archives of Clinical Neuropsychology (2000) | DNS was not described. |
| 116 | A long-term follow-up study of serial magnetic resonance images in patients with delayed encephalopathy after acute carbon monoxide poisoning | Inagaki T et al. | Psychiatry and Clinical Neurosciences (1997) | Control groups were not eligible for comparison with intervention groups. |
| 117 | Computed tomography of the brain in acute carbon monoxide poisoning | Silver DAT et al. | Clinical Radiology (1996) | Control groups were not eligible for comparison with intervention groups. |
| 118 | A longitudinal study of 100 consecutive admissions for carbon monoxide poisoning to the Royal Adelaide Hospital | Gorman DF et al. | Anaesthesia and Intensive Care (1992) | DNS was not described. |
| 119 | Hematological parameters as early predictors of delayed neurological sequelae in acute carbon monoxide poisoning | Shahin M et al. | Ain Shams Journal of Forensic Medicine and Clinical Toxicology (2020) | Intervention did not meet the criteria related to the brain imaging. |

**Supplementary Table S2. References of included studies in this meta-analysis**

1. Kim JH, Durey A, H SB, *et al*. Predictive factors for acute brain lesions on magnetic resonance imaging in acute carbon monoxide poisoning. *Am J Emerg Med* 2020;38:1825-30.

2. Kokulu K, Mutlu H, Sert ET. Serum netrin-1 levels at presentation and delayed neurological sequelae in unintentional carbon monoxide poisoning. *Clin Toxicol* 2020:1-7.

3. Lee H, Kang H, Ko BS, *et al*. Initial creatine kinase level as predictor for delayed neuropsychiatric sequelae associated with acute carbon monoxide poisoning. *Am J Emerg Med* 2020;25:30127-3.

4. Nah S, Choi S, Kim HB, *et al*. Cerebral white matter lesions on diffusion-weighted images and delayed neurological sequelae after carbon monoxide poisoning: A prospective observational study. Diagnostics 2020;10:698.

5. Jeon SB, Sohn CH, Seo DW, *et al*. Acute Brain Lesions on Magnetic Resonance Imaging and Delayed Neurological Sequelae in Carbon Monoxide Poisoning. *JAMA Neurol* 2018;75:436-43.

6. Kim YS, Cha YS, Kim MS, *et al*. The usefulness of diffusion-weighted magnetic resonance imaging performed in the acute phase as an early predictor of delayed neuropsychiatric sequelae in acute carbon monoxide poisoning. *Hum Exp Toxicol* 2018;37:587-95.

7. Kitamoto T, Tsuda M, Kato M, *et al*. Risk factors for the delayed onset of neuropsychologic sequelae following carbon monoxide poisoning. *Acute Med Surg* 2016;3:315-9.

8. Park E, Ahn J, Min YG, *et al*. The usefulness of the serum s100b protein for predicting delayed neurological sequelae in acute carbon monoxide poisoning. *Clin Toxicol* 2012;50:183-8.

9. Du X, Gu H, Hao F, *et al*. Utility of brain CT for predicting delayed encephalopathy after acute carbon monoxide poisoning. *Exp Ther Med* 2019;17:2682-10. Liao SC, Mao YC, Yang KJ, *et al*. Targeting optimal time for hyperbaric oxygen therapy following carbon monoxide poisoning for prevention of delayed neuropsychiatric sequelae: A retrospective study. *J Neurol Sci* 2019;396:187-92.

11. Tianhong W, Yanli Z, Youquan G, *et al*. Clinical features and risk factors analysis fo delayed encephalopathy after acute carbon monoxide poisoning. *Acta Medica Mediterranea* 2018;34:1177-80.

12. Kudo K, Otsuka K, Yagi J, *et al*. Predictors for delayed encephalopathy following acute carbon monoxide poisoning. *BMC Emerg Med* 2014;14:3.

13. Yang KC, Ku HL, Wu CL, *et al*. Striatal dopamine transporter binding for predicting the development of delayed neuropsychological sequelae in suicide attempters by carbon monoxide poisoning: A SPECT study. *Psychiatry Res Neuroimaging* 2011;194:219-23.

14. Ku HL, Yang KC, Lee YC, *et al*. Predictors of carbon monoxide poisoning-induced delayed neuropsychological sequelae. *Gen Hosp Psychiatry* 2010;32:310-4.

15. Ide T, Kamijo Y. The early elevation of interleukin 6 concentration in cerebrospinal fluid and delayed encephalopathy of carbon monoxide poisoning. Am *J Emerg Med* 2009;27:992-6.

16. Gaballah SZ, Elkhishin IAR, Hashim NA, *et al*. Predictors of Delayed Neurological Sequelae after Acute Carbon Monoxide Poisoning at Zagazig University Hospitals. *Zagazig J Forensic Med & Toxicology* 2020;18:105-21.

17. Lin MS, Lin CC, Yang CC, *et al*. Myocardial injury was associated with neurological sequelae of acute carbon monoxide poisoning in Taiwan. *J Chin Med Assoc* 2018;81:682-90.

**Supplementary Table S3. Detailed quality assessment of included studies using Quality Assessment of Diagnostic Accuracy Studies 2 (QUADAS-2)**

| QUADAS-II | | | | | | | | | | | | | | | | | | | |
| --- | --- | --- | --- | --- | --- | --- | --- | --- | --- | --- | --- | --- | --- | --- | --- | --- | --- | --- | --- |
| **Domain** | | MRI | | | | | | | | CT | | | | | | | No classification | | |
|  |  | 1 | 2 | 3 | 4 | 5 | 6 | 7 | 8 | 9 | 10 | 11 | 12 | 13 | 14 | 15 | 16 | 17 |  |
|  |  | Kim 2020 | Kokulu 2020 | Lee 2020 | Nah 2020 | Jeon 2018 | Kim 2018 | Kitamoto 2016 | Park 2012 | Du 2019 | Liao 2019 | Tianhong 2018 | Kudo 2014 | Yang 2011 | Ku 2010 | Ide 2009 | Gaballah 2020 | Lin 2018 |  |
| **Patient selection** | |  | | | | | | | |  | | | | | | |  | | |
| Risk of bias | 1 | low | low | low | low | low | low | low | high | low | low | low | low | high | low | low | low | low |  |
|  | 2 | low | low | low | low | low | low | low | low | low | low | low | low | low | low | low | low | low |  |
|  | 3 | low | low | low | low | low | low | low | low | low | low | low | low | low | low | low | low | low |  |
|  | **Total** | **low** | **low** | **low** | **low** | **low** | **low** | **low** | **high** | **low** | **low** | **low** | **low** | **high** | **low** | **low** | **low** | **low** |  |
| Applicability concerns | **Total** | **low** | **low** | **low** | **low** | **low** | **low** | **unclear** | **unclear** | **low** | **low** | **unclear** | **unclear** | **high** | **unclear** | **low** | **low** | **low** |  |
| **Index test** | |  | | | | | | | |  | | | | | | |  | | |
| Risk of bias | 1 | low | low | low | low | low | low | low | low | low | Low | low | low | low | low | low | low | Low |  |
|  | 2 | high | unclear | low | low | low | low | low | high | low | unclear | unclear | unclear | high | unclear | unclear | unclear | unclear |  |
|  | **Total** | **high** | **unclear** | **unclear** | **low** | **low** | **low** | **low** | **high** | **low** | **unclear** | **unclear** | **unclear** | **high** | **unclear** | **unclear** | **unclear** | **unclear** |  |
| Applicability concerns | **Total** | **low** | **low** | **unclear** | **low** | **low** | **low** | **unclear** | **unclear** | **low** | **unclear** | **unclear** | **unclear** | **unclear** | **unclear** | **unclear** | **unclear** | **unclear** |  |
| **Reference standard** | |  | | | | | | | |  | | | | | | |  | | |
| Risk of bias | 1 | high | low | low | low | low | low | low | low | low | low | low | high | low | low | low | unclear | low |  |
|  | 2 | unclear | unclear | unclear | unclear | unclear | unclear | unclear | unclear | unclear | unclear | unclear | unclear | unclear | unclear | unclear | unclear | unclear |  |
|  | **Total** | **high** | **low** | **low** | **low** | **low** | **low** | **low** | **low** | **low** | **low** | **low** | **high** | **low** | **low** | **low** | **unclear** | **low** |  |
| Applicability concerns | **Total** | **unclear** | **low** | **low** | **low** | **low** | **low** | **low** | **unclear** | **unclear** | **low** | **unclear** | **unclear** | **low** | **low** | **low** | **unclear** | **low** |  |
| **Flow and timing** | |  | | | | | | | |  | | | | | | |  | | |
| Risk of bias | 1 | low | low | low | low | low | low | low | unclear | low | unclear | low | low | low | low | low | low | low |  |
|  | 2 | unclear | low | low | low | low | low | low | low | low | low | low | low | low | low | low | low | low |  |
|  | 3 | unclear | low | low | low | low | low | low | unclear | low | low | low | low | low | low | low | low | low |  |
|  | **Total** | **high** | **low** | **low** | **low** | **low** | **low** | **low** | **high** | **Low** | **unclear** | **low** | **low** | **low** | **low** | **low** | **low** | **low** |  |
| **Final assessment** | | **High Bias** | **Low Bias** | **Low Bias** | **Low Bias** | **Low Bias** | **Low Bias** | **Low Bias** | **High Bias** | **Low Bias** | **High Bias** | **High Bias** | **High Bias** | **High Bias** | **High Bias** | **Low Bias** | **High Bias** | **Low Bias** |  |

**The Quality Assessment on Diagnostic Accuracy Studies (QUADAS-2)**

| **MAIN** | **PATIENT SELECTION** | **INDEX TEST** | **REFERENCE STANDARD** | **FLOW AND TIMING** |
| --- | --- | --- | --- | --- |
| **Description** | Describe methods of patient selection: Describe included patients (prior testing, presentation, intended use of index test and setting): | Describe the index test and how it was conducted and interpreted: | Describe the reference standard and how it was conducted and interpreted: | Describe any patients who did not receive the index test(s) and/or reference standard or who were excluded from the 2x2 table (refer to flow diagram): Describe the time interval and any interventions between index test(s) and reference standard: |
| **Signalling questions(yes/no/unclear)** | Was a consecutive or random sample of patients enrolled? | Were the index test results interpreted without knowledge of the results of the reference standard? | Is the reference standard likely to correctly classify the target condition? | Was there an appropriate interval between index test(s) and reference standard? |
|  | Was a case-control design avoided? | If a threshold was used, was it pre-specified? | Were the reference standard results interpreted without knowledge of the results of the index test? | Did all patients receive a reference standard? |
|  | Did the study avoid inappropriate exclusions? |  |  | Did all patients receive the same reference standard? |
|  |  |  |  | Were all patients included in the analysis? |
| **Risk of bias: High/low/unclear** | Could the selection of patients have introduced bias? | Could the conduct or interpretation of the index test have introduced bias? | Could the reference standard, its conduct, or its interpretation have introduced bias? | Could the patient flow have introduced bias? |
| **Concerns regarding applicability: High/low/unclear** | Are there concerns that the included patients do not match the review question? | Are there concerns that the index test, its conduct, or interpretation differ from the review question? | Are there concerns that the target condition as defined by the reference standard does not match the review question? |  |

**Reference**

Whiting PF, Rutjes AW, Westwood ME, *et al*. QUADAS-2: a revised tool for the quality assessment of diagnostic accuracy studies. *Ann Intern Med* 2011;155:529-36.

**Supplementary Table S4. Detailed statistical analysis using meta-disc software**

**MRI**

**Summary Sensitivity**

Study | Sen [95% Conf. Iterval.] TP/(TP+FN) TN/(TN+FP)

--------------------------------------------------------------------------------------------

Kim_2020 | 0.778 0.400 - 0.972 7/9 32/40

Kokulu_2020 | 0.722 0.584 - 0.835 39/54 112/129

Lee_2020 | 0.583 0.277 - 0.848 7/12 110/126

Nah_2020 | 0.600 0.406 - 0.773 18/30 93/124

Jeon_2018 | 0.752 0.657 - 0.833 76/101 258/286

Kim_2018 | 0.700 0.348 - 0.933 7/10 74/92

Kitamoto_2016 | 0.636 0.308 - 0.891 7/11 59/69

Park_2012 | 0.700 0.348 - 0.933 7/10 49/61

--------------------------------------------------------------------------------------------

**Pooled Sen | 0.709 0.647 - 0.766**

--------------------------------------------------------------------------------------------

Heterogeneity chi-squared = 3.98 (d.f.= 7) p = 0.782

Inconsistency (I-square) = 0.0 %

No. studies = 8.

Filter OFF

Add 1/2 to all cells of the studies with zero

**Summary Specificity**

Study | Spe [95% Conf. Iterval.] TP/(TP+FN) TN/(TN+FP)

--------------------------------------------------------------------------------------------

Kim_2020 | 0.800 0.644 - 0.909 7/9 32/40

Kokulu_2020 | 0.868 0.797 - 0.921 39/54 112/129

Lee_2020 | 0.873 0.802 - 0.926 7/12 110/126

Nah_2020 | 0.750 0.664 - 0.823 18/30 93/124

Jeon_2018 | 0.902 0.862 - 0.934 76/101 258/286

Kim_2018 | 0.804 0.709 - 0.880 7/10 74/92

Kitamoto_2016 | 0.855 0.750 - 0.928 7/11 59/69

Park_2012 | 0.803 0.682 - 0.894 7/10 49/61

--------------------------------------------------------------------------------------------

**Pooled Spe | 0.849 0.824 - 0.871**

--------------------------------------------------------------------------------------------

Heterogeneity chi-squared = 19.18 (d.f.= 7) p = 0.008

Inconsistency (I-square) = 63.5 %

No. studies = 8.

Filter OFF

Add 1/2 to all cells of the studies with zero

**Summary Positive Likelihood Ratio (Random effects model)**

Study | LR+ [95% Conf. Iterval.] % Weight

--------------------------------------------------------------------------------------------

Kim_2020 | 3.889 1.909 - 7.921 10.26

Kokulu_2020 | 5.480 3.416 - 8.793 14.15

Lee_2020 | 4.594 2.370 - 8.906 10.98

Nah_2020 | 2.400 1.573 - 3.661 15.07

Jeon_2018 | 7.686 5.313 - 11.118 16.03

Kim_2018 | 3.578 2.003 - 6.389 12.29

Kitamoto_2016 | 4.391 2.123 - 9.081 10.04

Park_2012 | 3.558 1.859 - 6.812 11.17

--------------------------------------------------------------------------------------------

**(REM) pooled LR+ | 4.278 3.093 - 5.918**

--------------------------------------------------------------------------------------------

Heterogeneity chi-squared = 19.66 (d.f.= 7) p = 0.006

Inconsistency (I-square) = 64.4 %

Estimate of between-study variance (Tau-squared) = 0.1354

No. studies = 8.

Filter OFF

Add 1/2 to all cells of the studies with zero

**Summary Negative Likelihood Ratio (Random effects model)**

Study | LR- [95% Conf. Iterval.] % Weight

--------------------------------------------------------------------------------------------

Kim_2020 | 0.278 0.081 - 0.952 2.59

Kokulu_2020 | 0.320 0.207 - 0.494 20.75

Lee_2020 | 0.477 0.244 - 0.935 8.69

Nah_2020 | 0.533 0.340 - 0.836 19.42

Jeon_2018 | 0.274 0.195 - 0.386 33.57

Kim_2018 | 0.373 0.144 - 0.966 4.34

Kitamoto_2016 | 0.425 0.193 - 0.935 6.33

Park_2012 | 0.373 0.144 - 0.970 4.31

--------------------------------------------------------------------------------------------

**(REM) pooled LR- | 0.357 0.293 - 0.436**

--------------------------------------------------------------------------------------------

Heterogeneity chi-squared = 6.81 (d.f.= 7) p = 0.449

Inconsistency (I-square) = 0.0 %

Estimate of between-study variance (Tau-squared) = 0.0000

No. studies = 8.

Filter OFF

Add 1/2 to all cells of the studies with zero

**Summary Diagnostic Odds Ratio (Random effects model)**

Study | DOR [95% Conf. Iterval.] % Weight

--------------------------------------------------------------------------------------------

Kim_2020 | 14.000 2.428 - 80.732 6.92

Kokulu_2020 | 17.129 7.820 - 37.522 17.46

Lee_2020 | 9.625 2.725 - 33.993 10.87

Nah_2020 | 4.500 1.951 - 10.381 16.60

Jeon_2018 | 28.011 15.421 - 50.882 20.80

Kim_2018 | 9.593 2.256 - 40.783 9.10

Kitamoto_2016 | 10.325 2.548 - 41.846 9.52

Park_2012 | 9.528 2.142 - 42.385 8.73

--------------------------------------------------------------------------------------------

**(REM) pooled DOR | 12.091 7.115 - 20.549**

--------------------------------------------------------------------------------------------

Heterogeneity chi-squared = 13.56 (d.f.= 7) p = 0.060

Inconsistency (I-square) = 48.4 %

Estimate of between-study variance (Tau-squared) = 0.2592

No. studies = 8.

Filter OFF

Add 1/2 to all cells of the studies with zero

**CT**

**Summary Sensitivity**

Study | Sen [95% Conf. Iterval.] TP/(TP+FN) TN/(TN+FP)

--------------------------------------------------------------------------------------------

Du_2019 | 0.815 0.619 - 0.937 22/27 47/96

Liao_2019 | 0.583 0.432 - 0.724 28/48 173/231

Tianhong_2018 | 0.778 0.608 - 0.899 28/36 138/148

Kudo_2014 | 0.769 0.462 - 0.950 10/13 61/65

Yang_2011 | 0.600 0.147 - 0.947 3/5 6/15

Ku_2010 | 0.769 0.462 - 0.950 10/13 12/17

Ide_2009 | 1.000 0.158 - 1.000 2/2 8/9

--------------------------------------------------------------------------------------------

**Pooled Sen | 0.715 0.634 - 0.787**

--------------------------------------------------------------------------------------------

Heterogeneity chi-squared = 8.00 (d.f.= 6) p = 0.238

Inconsistency (I-square) = 25.0 %

No. studies = 7.

Filter OFF

Add 1/2 to all cells of the studies with zero

**Summary Specificity**

Study | Spe [95% Conf. Iterval.] TP/(TP+FN) TN/(TN+FP)

--------------------------------------------------------------------------------------------

Du_2019 | 0.490 0.386 - 0.594 22/27 47/96

Liao_2019 | 0.749 0.688 - 0.803 28/48 173/231

Tianhong_2018 | 0.932 0.879 - 0.967 28/36 138/148

Kudo_2014 | 0.938 0.850 - 0.983 10/13 61/65

Yang_2011 | 0.400 0.163 - 0.677 3/5 6/15

Ku_2010 | 0.706 0.440 - 0.897 10/13 12/17

Ide_2009 | 0.889 0.518 - 0.997 2/2 8/9

--------------------------------------------------------------------------------------------

**Pooled Spe | 0.766 0.729 - 0.800**

--------------------------------------------------------------------------------------------

Heterogeneity chi-squared = 88.60 (d.f.= 6) p = 0.000

Inconsistency (I-square) = 93.2 %

No. studies = 7.

Filter OFF

Add 1/2 to all cells of the studies with zero

**Summary Positive Likelihood Ratio (Random effects model)**

Study | LR+ [95% Conf. Iterval.] % Weight

--------------------------------------------------------------------------------------------

Du_2019 | 1.596 1.224 - 2.083 17.50

Liao_2019 | 2.323 1.676 - 3.221 17.24

Tianhong_2018 | 11.511 6.171 - 21.472 15.48

Kudo_2014 | 12.500 4.622 - 33.807 12.70

Yang_2011 | 1.000 0.438 - 2.285 13.98

Ku_2010 | 2.615 1.182 - 5.788 14.22

Ide_2009 | 5.556 1.168 - 26.432 8.88

--------------------------------------------------------------------------------------------

**(REM) pooled LR+ | 3.371 1.755 - 6.473**

--------------------------------------------------------------------------------------------

Heterogeneity chi-squared = 54.98 (d.f.= 6) p = 0.000

Inconsistency (I-square) = 89.1 %

Estimate of between-study variance (Tau-squared) = 0.6152

No. studies = 7.

Filter OFF

Add 1/2 to all cells of the studies with zero

**Summary Negative Likelihood Ratio (Random effects model)**

Study | LR- [95% Conf. Iterval.] % Weight

--------------------------------------------------------------------------------------------

Du_2019 | 0.378 0.167 - 0.856 15.17

Liao_2019 | 0.556 0.395 - 0.784 30.50

Tianhong_2018 | 0.238 0.129 - 0.440 20.70

Kudo_2014 | 0.246 0.091 - 0.665 11.73

Yang_2011 | 1.000 0.290 - 3.454 8.46

Ku_2010 | 0.327 0.116 - 0.924 11.03

Ide_2009 | 0.196 0.015 - 2.495 2.41

--------------------------------------------------------------------------------------------

**(REM) pooled LR- | 0.387 0.257 - 0.581**

--------------------------------------------------------------------------------------------

Heterogeneity chi-squared = 10.25 (d.f.= 6) p = 0.114

Inconsistency (I-square) = 41.5 %

Estimate of between-study variance (Tau-squared) = 0.1111

No. studies = 7.

Filter OFF

Add 1/2 to all cells of the studies with zero

**Summary Diagnostic Odds Ratio (Random effects model)**

Study | DOR [95% Conf. Iterval.] % Weight

--------------------------------------------------------------------------------------------

Du_2019 | 4.220 1.477 - 12.064 17.33

Liao_2019 | 4.176 2.188 - 7.970 19.38

Tianhong_2018 | 48.300 17.511 - 133.22 17.53

Kudo_2014 | 50.833 9.865 - 261.93 13.92

Yang_2011 | 1.000 0.127 - 7.893 11.64

Ku_2010 | 8.000 1.522 - 42.042 13.81

Ide_2009 | 28.333 0.858 - 935.93 6.38

--------------------------------------------------------------------------------------------

**(REM) pooled DOR | 9.524 3.301 - 27.480**

--------------------------------------------------------------------------------------------

Heterogeneity chi-squared = 26.71 (d.f.= 6) p = 0.000

Inconsistency (I-square) = 77.5 %

Estimate of between-study variance (Tau-squared) = 1.3994

No. studies = 7.

Filter OFF

Add 1/2 to all cells of the studies with zero

**Supplementary Table S5. Reference list of included studies in each subgroup**

| **Characteristics** | **N** | **Reference number*** | |
| --- | --- | --- | --- |
| **MRI** | | |  |
| *Sample size* | | |  |
| ≥100 | 5 | **2, 3, 4, 5, 6** | |
| <100 | 3 | **1, 7, 8** | |
| *Inclusion criteria* | | |  |
| Level of COHb (≥5% or ≥3%) | 5 | **2, 3, 4, 6, 8** | |
| Clinical sign and symptom | 3 | **1, 5, 7** | |
| *Timing of imaging examination* | | |  |
| ≤72 hours | 4 | **3, 4, 5, 6** | |
| Unclear | 4 | **1, 2, 7, 8** | |
| *Hyperbaric oxygen therapy* | | |  |
| ≥80% | 5 | **1, 3, 4, 5, 6** | |
| <80% | 3 | **2, 7, 8** | |
| *Quality of study* | | |  |
| High quality | 6 | **2, 3, 4, 5, 6, 7** | |
| Low quality | 2 | **1, 8** | |
| **CT** | | |  |
| *Sample size* | | |  |
| ≥100 | 3 | **9, 10, 11** | |
| <100 | 4 | **12, 13, 14, 15** | |
| *Inclusion criteria* | | |  |
| Level of COHb (≥5% or ≥3%) | 2 | **10, 13** | |
| Clinical sign and symptom | 5 | **9, 11, 12, 14, 15** | |
| *Timing of imaging examination* | | |  |
| ≤72 hours | 0 | **-** | |
| Unclear | 7 | **9, 10, 11, 12, 13, 14, 15** | |
| *Hyperbaric oxygen therapy* | | |  |
| ≥80% | 5 | **10, 11, 13, 14, 15** | |
| <80% | 2 | **9, 12** | |
| *Quality of study* | | |  |
| High quality | 2 | **9, 15** | |
| Low quality | 5 | **10, 11, 12, 13, 14** | |

***References of included studies**

1. Kim JH, Durey A, H SB, *et al*. Predictive factors for acute brain lesions on magnetic resonance imaging in acute carbon monoxide poisoning. *Am J Emerg Med* 2020;38:1825-30.

2. Kokulu K, Mutlu H, Sert ET. Serum netrin-1 levels at presentation and delayed neurological sequelae in unintentional carbon monoxide poisoning. *Clin Toxicol* 2020:1-7.

3. Lee H, Kang H, Ko BS, *et al*. Initial creatine kinase level as predictor for delayed neuropsychiatric sequelae associated with acute carbon monoxide poisoning. *Am J Emerg Med* 2020;25:30127-3.

4. Nah S, Choi S, Kim HB, *et al*. Cerebral white matter lesions on diffusion-weighted images and delayed neurological sequelae after carbon monoxide poisoning: A prospective observational study. Diagnostics 2020;10:698.

5. Jeon SB, Sohn CH, Seo DW, *et al*. Acute Brain Lesions on Magnetic Resonance Imaging and Delayed Neurological Sequelae in Carbon Monoxide Poisoning. *JAMA Neurol* 2018;75:436-43.

6. Kim YS, Cha YS, Kim MS, *et al*. The usefulness of diffusion-weighted magnetic resonance imaging performed in the acute phase as an early predictor of delayed neuropsychiatric sequelae in acute carbon monoxide poisoning. *Hum Exp Toxicol* 2018;37:587-95.

7. Kitamoto T, Tsuda M, Kato M, *et al*. Risk factors for the delayed onset of neuropsychologic sequelae following carbon monoxide poisoning. *Acute Med Surg* 2016;3:315-9.

8. Park E, Ahn J, Min YG, *et al*. The usefulness of the serum s100b protein for predicting delayed neurological sequelae in acute carbon monoxide poisoning. *Clin Toxicol* 2012;50:183-8.

9. Du X, Gu H, Hao F, *et al*. Utility of brain CT for predicting delayed encephalopathy after acute carbon monoxide poisoning. *Exp Ther Med* 2019;17:2682-10. Liao SC, Mao YC, Yang KJ, *et al*. Targeting optimal time for hyperbaric oxygen therapy following carbon monoxide poisoning for prevention of delayed neuropsychiatric sequelae: A retrospective study. *J Neurol Sci* 2019;396:187-92.

10. Liao SC, Mao YC, Hung YM, et al. Predictive Role of QTc Prolongation in Carbon Monoxide Poisoning-Related Delayed Neuropsychiatric Sequelae. *Biomed Res Int* 2018;2018:2543018.

11. Tianhong W, Yanli Z, Youquan G, *et al*. Clinical features and risk factors analysis fo delayed encephalopathy after acute carbon monoxide poisoning. *Acta Medica Mediterranea* 2018;34:1177-80.

12. Kudo K, Otsuka K, Yagi J, *et al*. Predictors for delayed encephalopathy following acute carbon monoxide poisoning. *BMC Emerg Med* 2014;14:3.

13. Yang KC, Ku HL, Wu CL, *et al*. Striatal dopamine transporter binding for predicting the development of delayed neuropsychological sequelae in suicide attempters by carbon monoxide poisoning: A SPECT study. *Psychiatry Res Neuroimaging* 2011;194:219-23.

14. Ku HL, Yang KC, Lee YC, *et al*. Predictors of carbon monoxide poisoning-induced delayed neuropsychological sequelae. *Gen Hosp Psychiatry* 2010;32:310-4.

15. Ide T, Kamijo Y. The early elevation of interleukin 6 concentration in cerebrospinal fluid and delayed encephalopathy of carbon monoxide poisoning. Am *J Emerg Med* 2009;27:992-6.

**Supplementary Table S6. PRISMA 2020 checklist**

| **Section and Topic** | **Item #** | **Checklist item** | **Location where item is reported** |
| --- | --- | --- | --- |
| **TITLE** | | |  |
| Title | 1 | Identify the report as a systematic review. | 1 |
| **ABSTRACT** | | |  |
| Abstract | 2 | See the PRISMA 2020 for Abstracts checklist. | 2 |
| **INTRODUCTION** | | |  |
| Rationale | 3 | Describe the rationale for the review in the context of existing knowledge. | 3 |
| Objectives | 4 | Provide an explicit statement of the objective(s) or question(s) the review addresses. | 3-4 |
| **METHODS** | | |  |
| Eligibility criteria | 5 | Specify the inclusion and exclusion criteria for the review and how studies were grouped for the syntheses. | 10-11 |
| Information sources | 6 | Specify all databases, registers, websites, organisations, reference lists and other sources searched or consulted to identify studies. Specify the date when each source was last searched or consulted. | 11 |
| Search strategy | 7 | Present the full search strategies for all databases, registers and websites, including any filters and limits used. | 11,TableS6 |
| Selection process | 8 | Specify the methods used to decide whether a study met the inclusion criteria of the review, including how many reviewers screened each record and each report retrieved, whether they worked independently, and if applicable, details of automation tools used in the process. | 11-12 |
| Data collection process | 9 | Specify the methods used to collect data from reports, including how many reviewers collected data from each report, whether they worked independently, any processes for obtaining or confirming data from study investigators, and if applicable, details of automation tools used in the process. | 12 |
| Data items | 10a | List and define all outcomes for which data were sought. Specify whether all results that were compatible with each outcome domain in each study were sought (e.g. for all measures, time points, analyses), and if not, the methods used to decide which results to collect. | 12 |
|  | 10b | List and define all other variables for which data were sought (e.g. participant and intervention characteristics, funding sources). Describe any assumptions made about any missing or unclear information. | 12 |
| Study risk of bias assessment | 11 | Specify the methods used to assess risk of bias in the included studies, including details of the tool(s) used, how many reviewers assessed each study and whether they worked independently, and if applicable, details of automation tools used in the process. | 12 |
| Effect measures | 12 | Specify for each outcome the effect measure(s) (e.g. risk ratio, mean difference) used in the synthesis or presentation of results. | 13 |
| Synthesis methods | 13a | Describe the processes used to decide which studies were eligible for each synthesis (e.g. tabulating the study intervention characteristics and comparing against the planned groups for each synthesis (item #5)). | 13 |
|  | 13b | Describe any methods required to prepare the data for presentation or synthesis, such as handling of missing summary statistics, or data conversions. | 13 |
|  | 13c | Describe any methods used to tabulate or visually display results of individual studies and syntheses. | 13 |
|  | 13d | Describe any methods used to synthesize results and provide a rationale for the choice(s). If meta-analysis was performed, describe the model(s), method(s) to identify the presence and extent of statistical heterogeneity, and software package(s) used. | 13 |
|  | 13e | Describe any methods used to explore possible causes of heterogeneity among study results (e.g. subgroup analysis, meta-regression). | 13 |
|  | 13f | Describe any sensitivity analyses conducted to assess robustness of the synthesized results. | 13 |
| Reporting bias assessment | 14 | Describe any methods used to assess risk of bias due to missing results in a synthesis (arising from reporting biases). | NA |
| Certainty assessment | 15 | Describe any methods used to assess certainty (or confidence) in the body of evidence for an outcome. | NA |
| **RESULTS** | | |  |
| Study selection | 16a | Describe the results of the search and selection process, from the number of records identified in the search to the number of studies included in the review, ideally using a flow diagram. | 4 |
|  | 16b | Cite studies that might appear to meet the inclusion criteria, but which were excluded, and explain why they were excluded. | TableS1 |
| Study characteristics | 17 | Cite each included study and present its characteristics. | 4 |
| Risk of bias in studies | 18 | Present assessments of risk of bias for each included study. | 4-5 |
| Results of individual studies | 19 | For all outcomes, present, for each study: (a) summary statistics for each group (where appropriate) and (b) an effect estimate and its precision (e.g. confidence/credible interval), ideally using structured tables or plots. | Table1 |
| Results of syntheses | 20a | For each synthesis, briefly summarise the characteristics and risk of bias among contributing studies. | 5 |
|  | 20b | Present results of all statistical syntheses conducted. If meta-analysis was done, present for each the summary estimate and its precision (e.g. confidence/credible interval) and measures of statistical heterogeneity. If comparing groups, describe the direction of the effect. | 5 |
|  | 20c | Present results of all investigations of possible causes of heterogeneity among study results. | 5-6 |
|  | 20d | Present results of all sensitivity analyses conducted to assess the robustness of the synthesized results. | 5-6 |
| Reporting biases | 21 | Present assessments of risk of bias due to missing results (arising from reporting biases) for each synthesis assessed. | NA |
| Certainty of evidence | 22 | Present assessments of certainty (or confidence) in the body of evidence for each outcome assessed. | NA |
| **DISCUSSION** | | |  |
| Discussion | 23a | Provide a general interpretation of the results in the context of other evidence. | 6 |
|  | 23b | Discuss any limitations of the evidence included in the review. | 9-10 |
|  | 23c | Discuss any limitations of the review processes used. | 9-10 |
|  | 23d | Discuss implications of the results for practice, policy, and future research. | 10 |
| **OTHER INFORMATION** | | |  |
| Registration and protocol | 24a | Provide registration information for the review, including register name and registration number, or state that the review was not registered. | 10 |
|  | 24b | Indicate where the review protocol can be accessed, or state that a protocol was not prepared. | 10 |
|  | 24c | Describe and explain any amendments to information provided at registration or in the protocol. | 10 |
| Support | 25 | Describe sources of financial or non-financial support for the review, and the role of the funders or sponsors in the review. | 20 |
| Competing interests | 26 | Declare any competing interests of review authors. | 20 |
| Availability of data, code and other materials | 27 | Report which of the following are publicly available and where they can be found: template data collection forms; data extracted from included studies; data used for all analyses; analytic code; any other materials used in the review. | 13 |

**Supplementary Table S7. Search strategy**

| **Database** | **Search Term** | **N** |
| --- | --- | --- |
| **Medline**  (searching by OVID interface in Dec 26, 2020) | #1. exp Carbon Monoxide/ OR exp Carbon Monoxide Poisoning/ OR carbon monoxide poisoning.ti,ab,kw. OR carbon monoxide intoxication.ti,ab,kw. OR CO poisoning.ti,ab,kw. OR CO intoxication.ti,ab,kw.  #2. exp Cognition/ or exp Nervous System Diseases/ or exp Cognition Disorders/ OR exp Cognitive Dysfunction/ OR delayed neuropsychological sequelae.ti,ab,kw. OR delayed neurological sequelae.ti,ab,kw. OR DNS.ti,ab,kw.  #1 AND #2 | 22,676  2,546,568  1,226 |
| **Embase**  (searching by EMBASE interface in Dec 26, 2020) | #1. 'carbon monoxide'/exp OR 'carbon monoxide intoxication'/exp OR 'carbon monoxide':ab,kw,ti OR 'carbon monoxide intoxication':ab,kw,ti OR 'carbon monoxide poisoning':ab,kw,ti OR 'co poisoning':ab,kw,ti OR 'co intoxication':ab,kw,ti  #2. 'neurologic disease'/exp OR 'neuropsychology'/exp OR 'cognition'/exp OR 'cognitive defect'/exp OR 'delayed neurotoxicity'/exp OR 'delayed neurological sequelae':ab,kw,ti OR 'delayed neuropsychological sequelae':ti,ab,kw OR 'dns':ti,ab,kw  #1 AND #2 | 58,713  5,990,211  5,044 |
| **Cochrane library**  (searching by Cochrane interface in Dec 26, 2020) | #1. Carbon Monoxide [MeSH] OR Carbon Monoxide Poisoning [MeSH] OR carbon monoxide poisoning OR carbon monoxide intoxication  #2. Connition [MeSH] OR Nervous System Diseases [MeSH] OR Cognition Disorders [MeSH] OR delayed neurological sequelae OR delayed neuropsychological sequelae  #1 AND #2 | 668  93,223  51 |
| **Total** |  | 6,321 |
